# Isolation, purification and identification of biological compounds from *Beauveria* sp. and their evaluation as insecticidal effectiveness against *Bemisia tabaci*

**DOI:** 10.1038/s41598-021-91574-9

**Published:** 2021-06-08

**Authors:** Ran An, Maqsood Ahmed, Haiyan Li, Yanbin Wang, Aimin Zhang, Yuhui Bi, Zhiguo Yu

**Affiliations:** 1 College of Plant Protection, Shenyang Agricultural University, Shenyang, 110866 China; 2 Department of Agriculture, Pest Warning & Quality Control of Pesticides, Gujrat, 50700 Pakistan

**Keywords:** Chemistry, Chemical biology, Environmental chemistry

## Abstract

*Bemisia tabaci* is one of the most notorious agricultural pests in the world. A vicious circle among insect resistance, dose increased, environment and human body impaired as the overuse of synthetic pesticides are becoming increasingly evident. Entomopathogenic *Beauveria* sp. is known as an effective natural enemy to control *B. tabaci*. Therefore, this study aimed to purify and identify the biological compounds from *Beauveria* sp. LY2 via extensive chromatographic techniques, NMR and MS and evaluated for their insecticidal activities against *B. tabaci* via contact and feeding assay. The outcome identified that one new cerebroside, cerebroside F (**1**), nine known compounds, cerebroside B (**2**), bassiatin (**3**), methyl 1,4-dihydro-4-oxo-2-quinolinecarboxylate (**4**), cerevisterol (**5**), 9-hydroxycerevisterol (**6**), 6-dehydrocerevisterol (**7**), (*22*E,*24*R)-ergosta-8(14),22-diene-3β,5α,6β,7α-tetrol (**8**), melithasterol B (**9**) and ergosterol peroxide (**10**) were isolated. Among the known compounds, methyl 1,4-dihydro-4-oxo- 2-quinolinecarboxylate (**4**) was isolated from natural origin for the first time. It is demonstrable from the results that compounds **3**, **4** and **7** strongly featured insecticidal activities against *B. tabaci*, being the LC_50_ value as 10.59, 19.05, 26.59 μg/mL respectively in contact as well as 11.42, 5.66, 5.65 μg/mL respectively in feeding experiment. Moreover, no adverse effect on plant growth/height or phytotoxicity was observed on pepper, cucumber, tomato and cotton. The data from the current study has provided the foundation for the use of newly purified compounds against *Bemisia tabaci* as an alternative to synthetic chemical compounds.

## Introduction

The whitefly, *Bemisia tabaci* (Hemiptera: Aleyrodidae) is one of the world’s top 100 invasive species which can attack more than 600 agricultural plant-host species under field and greenhouse conditions^1, 2^. Due to its piercing-sucking mouthpart, *B. tabaci* can directly cause plant weakness and indirectly transmit approximately 111 plant viruses^3^. Both nymphs and adults can also secrete honeydew, which can induce coal pollution. Besides B biotype (MEAM1) is the dominant species which significantly threatened many agricultural commodities in numerous countries^4–6^. Moreover, for the excessive use of synthetic pesticides have contributed to resistance development of *B. tabaci*. So far, 64 active ingredients have been reported for resistance from *B. tabaci*^7^.

The injudicious and excessive use of broad-spectrum synthetic pesticide can do harm to human health and environment^8^, and it can also result in the development of heritable resistance, pest resurgence and secondary pest problems. In contrast, bio-insecticides are safer for humans and more environmentally friendly. Moreover, plant and fungi are the sources of thousands of secondary metabolites^9^. Those secondary metabolites are ideal substitutes of those chemicals which play a significant role and possess a great potential on the field of biopesticides^10^.

*Beauveria* sp. (Ascomycota: Hypocreales) is a facultative entomopathogen with an extremely broad spectrum which is used as a commercial biopesticide against many agriculturally important insect pests^11^. The metabolites from *Beauveria* sp. can be divided into three main kinds, such as alkaloids (tenellin, bassiatin, pyridovercin, pyridomacrolidin, ilicicdin H), cyclodepsipetides (beauvericins, allobeauvericins, bassianolides, beauveriolides) and benzoquinone (oosporein), many of them exert insecticidal^12, 13^, anthelminthic^14^, synergistic antifungal^15^, antibacterial^16, 17^, antiviral^18^ and cytotoxic activities^19^. However, research on the secondary metabolites of *Beauveria* sp. has a history of nearly 60 years, but the research on its activity is mainly focused on the field for medicinal use, and there are very few activities in the field of agriculture, in particular insecticidal activity. Although some studies have been conducted in isolation and identification of various compounds from natural resources, but, there is no research of bassiatin in the agricultural field per se. Therefore, this research not only supplements the gap in the insecticidal activity of bassiatin, but also adds two types of substances to the group of secondary metabolites of *Beauveria* sp., and evaluates their insecticidal effects in both feeding and contact toxicity effects.

## Results

### Extraction, separation and purification of extract

**Figure 1:**
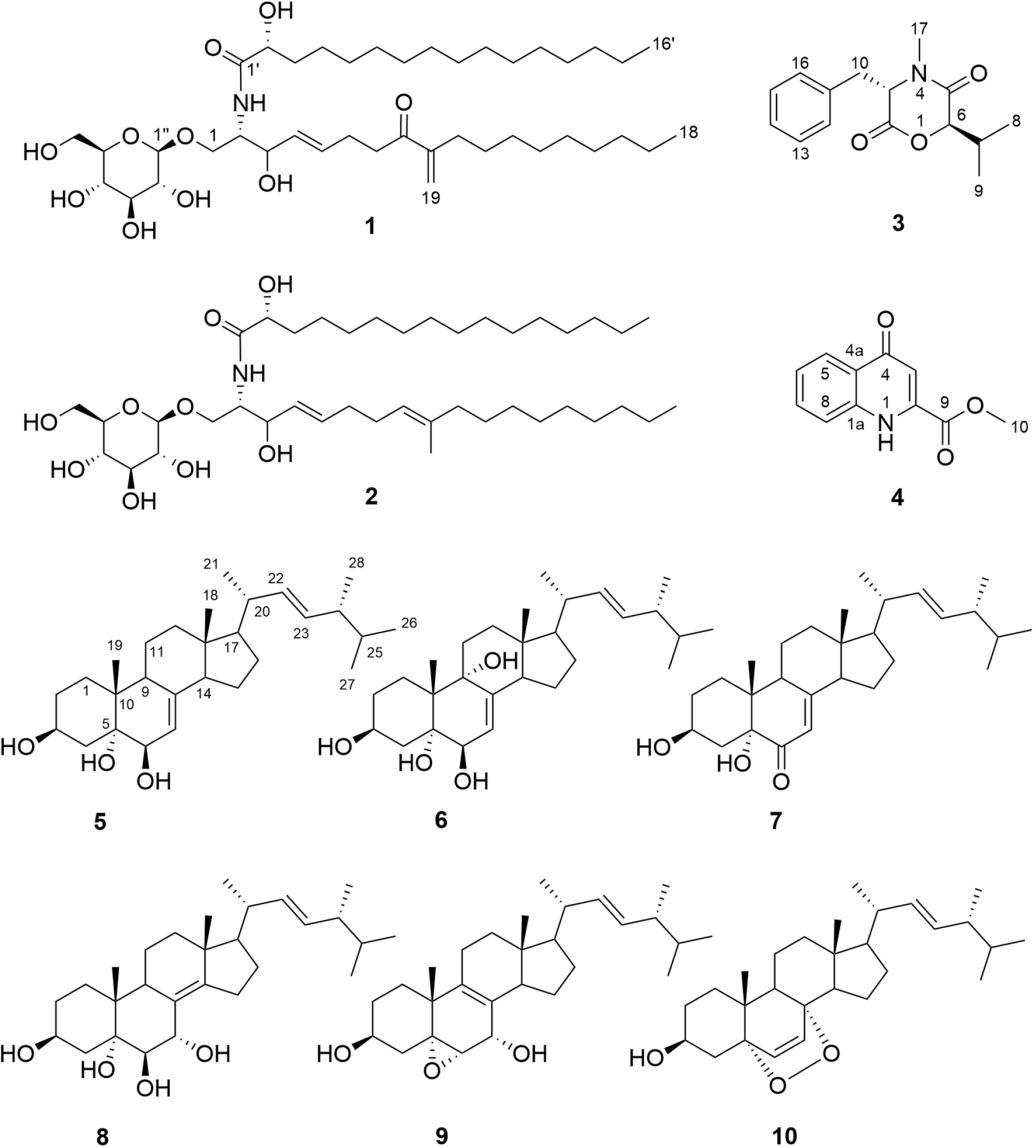
The chemical structures of the compounds **1**–**10.**

After silica gel column chromatography, gel chromatography and HPLC, compounds **1**–**10** were obtained. Among them, compound **1** is a new cerebroside compound, **4** is a new natural product, and **2**–**10** are all confirmed to be known compounds after comparison with previous studies, they are cerebroside B (**2**)^20^, bassiatin (**3**)^21^, methyl 1,4-dihydro-4-oxo-2-quinolinecarboxylate (**4**)^22, 23^, cerevisterol (**5**)^24^, 9-hydroxycerevisterol (**6**)^25^, 6-dehydrocerevisterol (**7**)^26^, (22*E*,24*R*)-ergosta-8(14),22-diene-3β,5α,6β,7α-tetrol (**8**)^27^, melithasterol B (**9**)^28^, ergosterol peroxide (**10**)^28^. Compounds **1**–**2**, **4**–**9** are all isolated from *Beauveria* sp. for the first time. The chemical structures of all the purified compounds (1–10) are presented in Fig. 1.

### Structure elucidation of compounds

**Figure 2:**
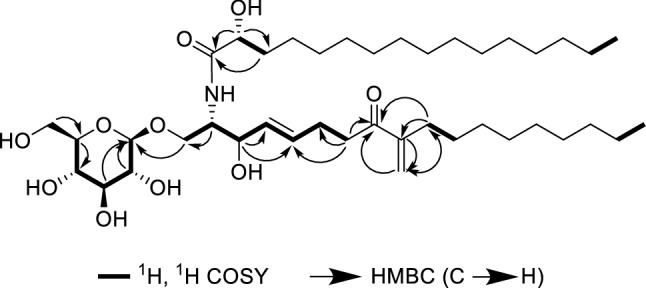
The key COSY (bold) and HMBC (arrows) correlations of compound **1.**

Compound **1**, white amorphous powder, possessed a molecular formula of C_41_H_75_NO_10_ by the positive HR-ESI-MS (m/z 742.5457 [M+H]^+^, calcd. 742.5469), requiring five indices of hydrogen deficiency. The ^1^H NMR spectrum (Table 1) in methanol-*d*_4_ of **1** showed two terminal olefinic protons at δ_H_ 5.79, 6.09 (each 1H, br s, H-19), two mutually coupled trans olefinic protons at δ_H_ 5.74 (1H, dtd, *J* = 15.4, 6.6, 0.7 Hz, H-5), 5.50 (1H, ddt, *J* = 15.4, 7.3, 1.3 Hz, H-4), the diagnostic anomeric proton of β-glucopyranoside moiety at δ_H_ 4.26 (1H, d, *J* = 7.8 Hz, H-1″), as well as two long-chain terminal methyl triplets at δ_H_ 0.89 (6H, t, *J* = 7.0 Hz, H-18, H-16′). The ^13^C NMR spectrum (Table 1) exhibited an α,β-unsaturated carbonyl carbon at δ_C_ 203.0 (s, C-8), an amide acyl carbon at δ_C_ 177.2 (s, C-1′), four olefinic carbon signals, a set of characteristic carbons corresponding to a β-D-glucopyranoside moiety appeared at δ_C_ 104.7 (d, C-1″), 75.0 (d, C-2″), 77.9 (d, C-3″), 71.6 (d, C-4″), 78.0 (d, C-5″) and 62.7 (t, C-6″), other three oxygenated carbons at δ_C_ 73.0 (d), 72.7 (d) and 69.7 (t), as well as a series of long-chain aliphatic carbon signals. The above NMR features were similar to those of cerebroside B (**2**)^20^, also isolated from the current study, and the obvious difference between them only came from the olefinic methyl part of the main chain, in which the olefinic methyl signal was removed and replaced by a terminal olefinic methylene in **1**, in addition, a keto carbonyl at δ_C_ 203.0 was newly detected. In the HMBC spectrum (Figure S3), the correlations from H-19 [δ_H_ 5.79, 6.09 (each 1H, br s)] to the keto carbonyl carbon confirmed the presence of α, β-unsaturated carbonyl moiety. Furthermore, the correlations from H-7 [2.82 (2H, br t, *J* = 7.4 Hz)] to C-5 [133.4 (d)] and C-8 [203.0 (s)] revealed the keto carbonyl at C-8. In view of their consistency of the chemical shifts and coupling constants, the configurations of C-2, C-3 and C-2′ were deduced to be the same as those of **2**. The interpretation of the MS fragment ions (see Figure S8 in supplementary material) allowed to determine the specific length of the two chains. Therefore, the structure of compound **1** was established as illustrated in Fig. 2 and named as cerebroside F.

**Table 1:** The ^1^H NMR and ^13^C NMR spectral data of the compound **1** in methanol-*d4.*

No.	^1^H NMR	^13^C NMR	No.	^1^H NMR	^13^C NMR
1	3.70 (1H, dd, 10.4, 3.6) 4.10 (1H, dd, 10.4, 5.6)	69.7 (t)	19	5.79, 6.09 (each 1H, br s)	125.2 (t)
2	3.98 (1H, m)	54.6 (d)	1′	–	177.2 (s)
3	4.13 (1H, br dd, 7.5, 7.3)	72.7 (d)	2′	3.97 (1H, m)	73.0 (d)
4	5.50 (1H, ddt, 15.4, 7.3, 1.3)	131.7 (d)	3′	1.54, 1.71 (each 1H, m)	35.9 (t)
5	5.74 (1H, dtd, 15.4, 6.6, 0.7)	133.4 (d)	4′	1.35−1.45 (2H, m)	26.2 (t)
6	2.26−2.33 (2H, m)	28.1 (t)	5′−13′	1.24−1.33 (18H, m)	30.5−30.8 (t)
7	2.82 (2H, br t, 7.4)	38.1 (t)	14′	1.27 (2H, m)	33.1 (t)
8	–	203.0 (s)	15′	1.30 (2H, m)	23.7 (t)
9	–	150.2 (s)	16′	0.89 (3H, t, 7.0)	14.4 (q)
10	2.25 (2H, br t, 7.6)	32.0 (t)	1″	4.26 (1H, d, 7.8)	104.7 (d)
11	1.38 (2H, m)	29.8 (t)	2″	3.18 (1H, dd, 9.2, 7.8)	75.0 (d)
12−15	1.24−1.33 (8H, m)	30.5−30.8 (t)	3″	3.35 (1H, dd, 9.2, 8.8)	77.9 (d)
16	1.27 (2H, m)	33.1 (t)	4″	3.27 (1H, m)	71.6 (d)
17	1.30 (2H, m)	23.7 (t)	5″	3.27 (1H, m)	78.0 (d)
18	0.89 (3H, t, 7.0)	14.4 (q)	6″	3.66 (1H, dd, 11.9, 5.5) 3.86 (1H, dd, 11.9, 1.3)	62.7 (t)

Compound **2**, white amorphous powder, C_41_H_77_NO_9_, HR-ESI–MS (m/z 728.5625 [M + H]^+^). ^1^H NMR (s, 600 MHz, DMSO) δ 7.39 (d, *J* = 9.3 Hz, 1H, NH), 5.59 (m, 1H, H-5), 5.50 (d, *J* = 5.2 Hz, 1H, OH-2′), 5.40 (dd, *J* = 15.4, 6.8 Hz, 1H, H-4), 5.09 (d, *J* = 6.6 Hz, 1H, H-8), 4.96 (d, *J* = 4.1 Hz, 1H, OH-3), 4.93 (d, *J* = 5.5 Hz, 1H, OH-3″), 4.90 (dd, *J* = 7.9, 5.0 Hz, 2H, OH-2″, OH-4″), 4.51 (t, *J* = 5.9 Hz, 1H, OH-6″), 4.12 (d, *J* = 7.8 Hz, 1H, H-1″), 3.99 (dd, *J* = 12.9, 6.6 Hz, 1H, H-1), 3.92 (dd, *J* = 10.4, 5.8 Hz, 1H, H-3), 3.81 (m, 2H, H-2, H-2′), 3.66 (dd, *J* = 10.0, 6.1 Hz, 1H, H-6″), 3.52 (dd, *J* = 10.3, 3.7 Hz, 1H, H-1), 3.43 (dt, *J* = 11.7, 5.9 Hz, 1H, H-6″), 3.13 (td, *J* = 8.8, 4.8 Hz, 1H, H-3″), 3.08 (d, *J* = 5.8 Hz, 1H, H-5″), 3.04 (dd, *J* = 8.8, 5.2 Hz, H, H-4″), 2.95 (td, *J* = 8.4, 4.2 Hz, 1H, H-2″), 1.98 (m, 4H, H-6, H-7), 1.92 (t, *J* = 7.5 Hz, 2H, H-10), 1.55 (d, *J* = 14.5 Hz, 3H, Me-9), 1.47–1.14 (overlap, m, 40H, CH_2_-11 ~ 17, CH_2_-3′ ~ 15′), 0.86 (s, m, 6H, Me-18, Me-16′). ^13^C NMR (151 MHz, DMSO) δ 174.14 (C-1′), 135.26 (C-9), 131.47 (C-5), 131.39 (C-4), 123.86 (C-8), 103.89 (C-1″), 77.28 (C-5″), 76.93 (C-3″), 73.78 (C-2″), 71.41 (C-2′), 70.93 (C-1), 70.39 (C-4″), 69.11 (C-3), 61.44 (C-6″), 53.22 (C-2), 39.43 (C-10) 34.86 (C-3′), 32.54 (C-6), 31.69–29.07 (overlap, C-12 ~ 16, C-5′ ~ 14′), 27.75 (C-7), 27.62 (C-11), 24.92 (C-4′), 22.49 (C-15′), 16.09 (C-19), 14.31 (overlap, C-18, C-16′).

Compound **3**, colorless crystal, C_15_H_19_NO_3_, ESI–MS (m/z 261.00 [M]^+^) ^1^H NMR (600 MHz, CDCl_3_) δ 7.33 (overlap, m, 3H, H-13,H-14,H-15), 7.13 (overlap, m, 2H, H-12, H-16), 4.41 (t, *J* = 4.4 Hz, 1H, H-3), 3.24 (dd, *J* = 37.0, 4.4 Hz, 1H, H-10), 3.03 (s, 3H, N- Me), 3.01 (d, *J* = 2.2 Hz, 1H, H-6), 2.30 (hd, *J* = 6.9, 2.2 Hz, 1H, H-7), 0.85 (d, *J* = 7.1 Hz, 3H, Me-8), 0.77 (d, *J* = 6.8 Hz, 3H, Me-9). ^13^C NMR (151 MHz, CDCl_3_) δ 167.38 (s, C-2), 165.64 (s, C-5), 134.26 (s, C-11), 129.89 (overlap, s, C-12, C-16), 129.34 (overlap, s, C-13, C-15), 128.33 (s, C-14), 81.38 (s, C-6), 62.87 (s, C-3), 37.19 (s, C-10), 32.53 (s, N- Me), 29.77 (s, C-7), 18.68 (s, C-8), 15.24 (s, C-9).

Compound **4**, white amorphous powder, C_11_H_9_NO_3_, ESI-MS (m/z 202.20 [M-H]^-^). ^1^H NMR (600 MHz, CDCl_3_) δ 9.26 (s, 1H, H-N), 8.36 (d, *J* = 8.1 Hz, 1H, H-5), 7.67 (t, *J* = 7.7 Hz, 1H, H-7), 7.48 (d, *J* = 8.3 Hz, 1H, H-8), 7.39 (t, *J* = 7.6 Hz, 1H, H-6), 6.99 (s, 1H, H-3), 4.04 (s, 3H, H-10). ^13^C NMR (151 MHz, CDCl_3_) δ179.55 (C-4), 163.40 (C-9), 138.96 (C-1a), 136.10 (C-2), 133.10 (C-7), 126.34 (C-4a), 126.27 (C-5), 124.51 (C-6), 117.97 (C-8), 111.65 (C-3), 53.76 (C-10).

Compound **5**, white amorphous powder, C_28_H_46_O_3_, ESI–MS (m/z 431.20 [M + H]^+^). ^1^H NMR (600 MHz, CDCl_3_) δ 5.36 (m, 1H, H-7), 5.23 (dd, *J* = 15.3, 7.5 Hz, 1H, H-23), 5.16 (dd, *J* = 15.3, 8.2 Hz, 1H, H-22), 4.08 (td, *J* = 11.3, 5.7 Hz, 1H, H-3), 3.62 (d, *J* = 5.0 Hz, 1H, H-6), 1.09 (s, 3H, Me-19), 1.03 (d, *J* = 6.6 Hz, 3H, Me-21), 0.92 (d, *J* = 6.8 Hz, 3H, Me-28), 0.83 (dd, *J* = 9.3, 6.8 Hz, 6H Me-26, Me-27), 0.60 (s, 3H, Me-18). ^13^C NMR (151 MHz, CDCl_3_) δ144.03 (C-8), 135.39 (C-22), 132.20 (C-23), 117.56 (C-7), 75.98 (C-5), 73.69 (C-6), 67.74 (C-3), 56.01 (C-17), 54.77 (C-14), 43.78 (C-13), 43.49 (C-9), 42.83 (C-24), 40.40 (C-20), 39.49 (C-4), 39.24 (C-12), 37.16 (C-20), 33.09 (C-25), 32.98 (C-1), 30.88 (C-2), 27.91 (C-16), 22.90 (C-15), 22.06 (C-11), 21.13 (C-21), 19.96 (C-26), 19.65 (C-27), 18.85 (C-19), 17.60 (C-28), 12.34 (C-18).

Compound **6**, white amorphous powder, C_28_H_46_O_4_, HR-ESI–MS (m/z 915.6686 [2 M + Na]^+^). ^1^H NMR (600 MHz, MeOD) δ 5.36 (dd, *J* = 5.2, 2.3 Hz, 1H, H-7), 5.26 (m, 2H, H-22, H-23), 4.03 (m, 1H, H-3), 3.68 (dd, *J* = 5.2, 2.5 Hz, 1H, H-6), 2.53 (m, 1H, H-14), 1.15 (s, 3H, Me-19), 1.07 (t, *J* = 6.2 Hz, 3H, Me-21), 0.98 (t, *J* = 7.2 Hz, 3H, Me-28), 0.89 (dd, *J* = 11.6, 6.8 Hz, 6H, Me-26, Me-27), 0.69 (s, 3H, Me-18). ^13^C NMR (151 MHz, MeOD) δ 144.65 (C-8), 137.84 (C-22), 134.17 (C-23), 121.89 (C-7), 79.77 (C-5), 76.95 (C-9), 74.57 (C-6), 69.03 (C-3), 58.24 (C-17), 52.74 (C-14), 45.68 (C-13), 45.22 (C-24), 42.70 (C-20), 42.28 (C-10), 41.61 (C-4), 37.39 (C-12), 35.24 (C-25), 32.50 (C-2), 30.06 (C-11), 30.03(C-16), 29.12 (C-1), 24.87 (C-15), 23.09 (C-19), 22.50 (C-21), 21.33 (C-26), 20.95 (C-27), 19.07 (C-28), 13.13 (C-18).

Compound **7**, white amorphous powder, C_28_H_44_O_3_, ESI–MS (m/z 429.20 [M + H]^+^). ^1^H NMR (600 MHz, CDCl_3_) δ 5.65 (s, 1H, H-7), 5.24 (dd, *J* = 15.3, 7.7 Hz, 1H, H-23), 5.16 (dd, *J* = 15.3, 8.4 Hz, 1H, H-22), 4.03 (m, 1H, H-3), 2.52(m, 1H, H-13), 1.04 (d, *J* = 6.6 Hz, 3H, Me-21), 0.96 (d, *J* = 5.8 Hz, 3H, Me-19), 0.92 (d, *J* = 6.8 Hz, 3H, Me-28), 0.83 (dd, *J* = 9.6, 6.8 Hz, 6H, Me-27, Me-26), 0.61 (s, 3H, Me-18). ^13^C NMR (151 MHz, CDCl_3_) δ 198.31 (C-6), 165.32 (C-8), 135.02 (C-22), 132.51 (C-23), 119.71 (C-7), 77.81 (C-5), 67.50 (C-3), 56.05 (C-17), 55.82 (C-14), 44.78 (C-9), 43.90 (C-13), 42.82 (C-24), 40.46 (C-20), 40.28 (C-12), 38.85 (C-4), 36.51 (C-10), 33.06 (C-25), 30.37 (C-2), 30.23 (C-1), 29.71 (C-16), 27.86 (C-16), 22.49 (C-11), 21.96 (C-15), 21.11 (C-21), 19.95 (C-26), 19.65 (C-27), 17.58 (C-19), 16.43 (C-28), 12.70 (C-18).

Compound **8**, white amorphous powder, C_28_H_46_O_4_, ESI–MS (m/z 447.10 [M + H]^+^). ^1^H NMR (600 MHz, CDCl_3_) δ 5.20 (qd, *J* = 15.3, 7.3 Hz, 2H, H-22, H-23), 4.42 (s, 1H, H-6), 3.91(m, 1H, H-3), 3.14 (d, *J* = 3.5 Hz, 1H, H-7), 2.61 (dt, *J* = 17.0, 8.4 Hz, 1H, H-15), 1.02 (d, *J* = 6.7 Hz, 3H, Me-21), 0.91 (t, *J* = 7.3 Hz, 3H, Me-25), 0.87 (m, 6H, Me-18, Me-19), 0.83 (dd, *J* = 9.9, 6.8 Hz, 6H, Me-27, Me-28). ^13^C NMR (s, 151 MHz, CDCl_3_) δ152.62 (C-14), 135.27 (C-22), 132.26 (C-23), 125.19 (C-8), 68.69 (C-3), 67.80 (C-5), 65.08 (C-6), 61.35 (C-7), 56.83 (C-17), 42.98 (C-24), 42.85 (C-13), 39.58 (C-20), 39.25 (C-3), 38.74 (C-9), 36.60 (C-12), 35.85 (C-10), 33.10 (C-26), 32.20 (C-1), 31.11 (C-2), 27.18 (C-16), 24.96 (C-15), 21.24 (C-21), 19.97 (C-28), 19.67 (C-27), 19.00 (C-11), 18.07 (C-19), 17.62 (C-18), 16.53 (C-25).

Compound **9**, white amorphous powder, C_28_H_44_O_3_, ESI–MS (m/z 451.30 [M + Na]^+^). ^1^H NMR (600 MHz, CDCl_3_) δ 5.19 (dq, *J* = 15.3, 7.7 Hz, 2H, H-22, H-23), 4.22 (s, 1H, H-7), 3.95 (m, 1H, H-3), 3.31 (d, *J* = 2.6 Hz, 1H, H-6), 1.14 (s, 1H, H-19), 1.02 (d, *J* = 6.6 Hz, 3H, Me-21), 0.91 (d, *J* = 6.8 Hz, 3H, Me-28), 0.83 (dd, *J* = 9.5, 6.8 Hz, 6H, Me-26, Me-27), 0.59 (s, 3H, Me-18). ^13^C NMR (s, 151 MHz, CDCl_3_) δ 135.56 (C-22), 134.47 (C-9), 132.01 (C-23), 126.94 (C-8), 68.57 (C-3), 67.12 (C-7), 65.66 (C-5), 62.62 (C-6), 49.60 (C-14), 42.84 (C-24), 42.09 (C-13), 40.41 (C-20), 39.16 (C-4), 38.00 (C-10), 35.68 (C-12), 33.10(C-25), 30.84 (C-2), 30.20 (C-1), 29.01 (C-16), 23.85 (C-15), 23.4 1(C-11), 22.81 (C-19), 20.96 (C-21), 19.97 (C-27), 19.65 (C-26), 17.66 (C-28), 11.29 (C-18).

Compound **10**, white amorphous powder, C_28_H_44_O_3_, ESI–MS (m/z 429.20 [M + H]^+^). ^1^H NMR (600 MHz, CDCl_3_) δ 6.50 (d, *J* = 8.5 Hz, 1H, H-7), 6.24 (d, *J* = 8.5 Hz, 1H, H-6), 5.22 (dd, *J* = 15.2, 7.7 Hz, 1H, H-23), 5.14 (dd, *J* = 15.3, 8.4 Hz, 1H, H-22), 3.97 (m, 1H, H-3), 1.00 (d, *J* = 6.6 Hz, 3H, Me-21), 0.91 (d, *J* = 6.8 Hz, 3H, Me-28), 0.88 (s, 3H, Me-19), 0.83 (d, *J* = 6.8 Hz, 3H, Me-27), 0.82 (overlap, m, 6H, Me-18, Me-26). ^13^C NMR (151 MHz, CDCl_3_) δ 135.44 (s, C-6), 135.22 (s, C-22), 132.32 (s, C-23), 130.76 (s, C-7), 82.17 (s, C-5), 79.44 (s, C-8), 66.46 (s, C-3), 56.20 (s, C-17), 51.69 (s, C-14), 51.09 (s, C-9), 44.57 (s, C-13), 42.78 (s, C-24), 39.74 (s, C-20), 39.35 (s, C-12), 36.97 (s, C-4), 36.93 (s, C-10), 34.70 (s, C-1), 33.07 (s, C-25), 30.11 (s, C-2), 28.65 (s, C-16), 23.40 (s, C-11), 20.88 (s, C-21), 20.63 (s, C-15), 19.95 (s, C-27), 19.65 (s, C-26), 18.18 (s, C-19), 17.56 (s, C-28), 12.87 (s, C-18).

### In-vitro insecticidal activity of isolated compounds

**Figure 3:**
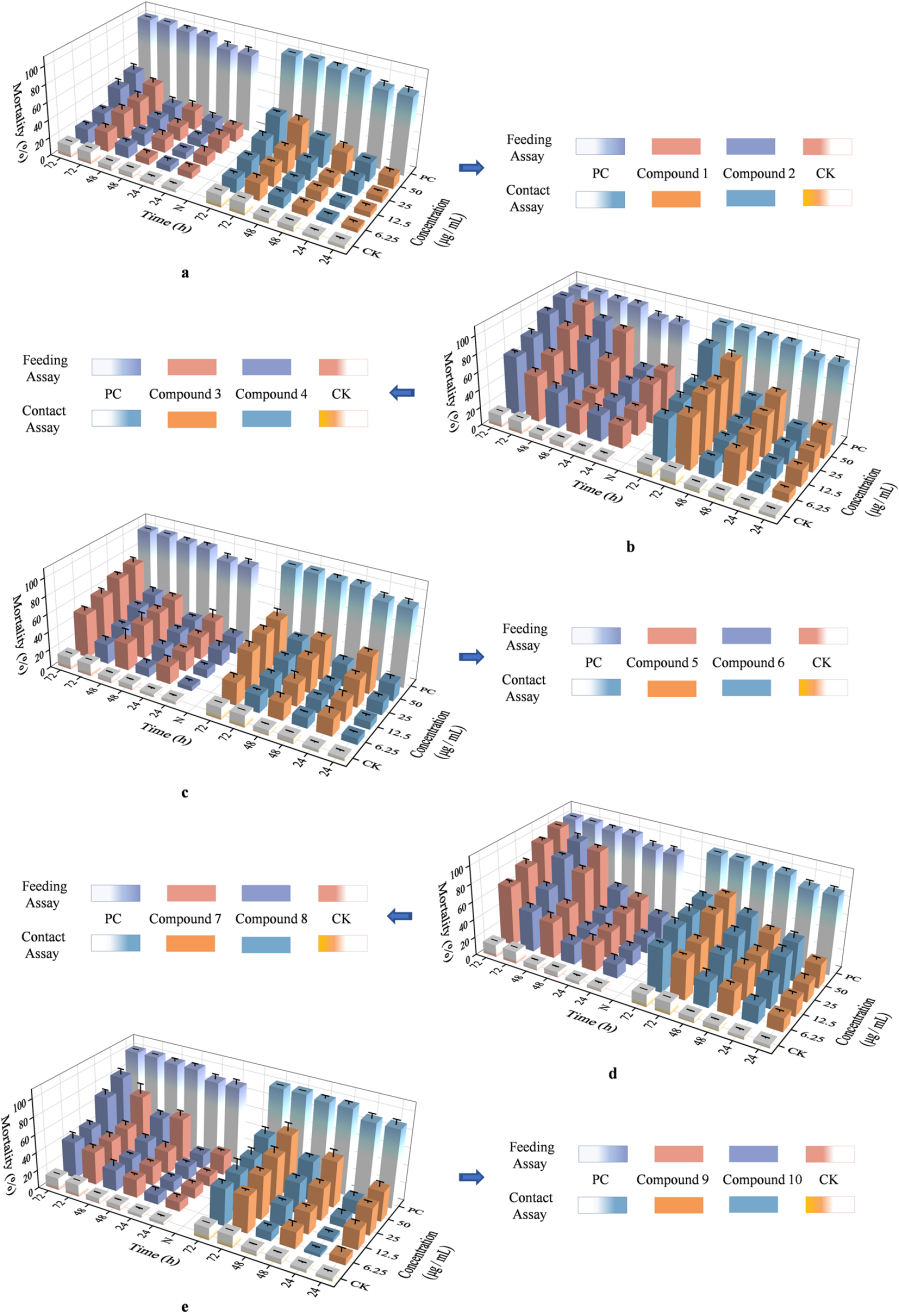
The morality rate of compounds **1–10** with different concentrations and assay methods at 24 h, 48 h and 72 h.

After the isolation, purification and identification, compounds **1**–**10** were evaluated for the insecticidal activity against *B. tabaci.* The results of the mean mortality via both contact and feeding toxicity was exhibited in Fig. 3. Futhermore, the results of the LC_50_ value at 72 h were presented in Table 2. However, detailed data was placed in supplementary material as Tables S1 to S10.

**Table 2:** Probit analysis of the effects of compounds **1–10** on *B. tabaci* at 72 h.

	Compound	LC_50_ μg/mL	95% F.L		Slope ± SE	X^2^
			Lower	Upper		
Contact Assay	1	> 50	36.33	777.49	0.96 ± 0.34	0.42
	2	> 50	40.42	265.48	0.99 ± 0.21	0.05
	3	10.59	4.74	23.64	0.92 ± 0.18	1.02
	4	19.05	13.74	26.40	0.88 ± 0.07	5.60
	5	> 50	22.75	313.89	0.89 ± 0.29	2.03
	6	> 50	22.83	3788.71	1.00 ± 0.57	0.01
	7	26.59	16.80	42.07	0.96 ± 0.10	0.83
	8	> 50	6.87	593.56	0.95 ± 0.49	0.18
	9	41.98	20.34	84.76	0.99 ± 0.16	0.10
	10	> 50	13.90	1548.93	0.92 ± 0.53	0.42
Feeding Assay	1	> 50	6.82	52764.85	0.95 ± 0.99	0.24
	2	> 50	36.52	228.20	0.98 ± 0.20	0.72
	3	11.42	8.51	15.34	0.99 ± 0.07	0.74
	4	5.66	3.38	9.47	0.99 ± 0.11	0.37
	5	22.29	12.35	40.24	0.99 ± 0.13	0.17
	6	> 50	23.66	1704.02	0.98 ± 0.47	0.34
	7	5.65	3.38	9.47	0.99 ± 0.11	0.36
	8	13.83	10.32	18.53	0.99 ± 0.06	1.06
	9	> 50	27.75	149.46	0.81 ± 0.19	3.30
	10	22.06	16.36	29.74	0.97 ± 0.07	1.25

F.L (Fiducial Limit); X^2^; (Chi-square); SE (Standard Error); LC_50_ (Lethal Concentration).

According to the mortality results of compound **1**, 41.67% mortality was reported at 72 h at 50 μg/mL via contact assay. Similarly, 38.33% mortality was displayed at the same concentration and time exposure via feeding assay (Fig. 3a). Similar results for insecticidal activity were afforded by compound **2** by both bioassays i.e. 47.50% and 44.17% mortality was recorded at 50 μg/mL via contact as well as feeding assay respectively (Fig. 3a). Results apparent from Fig. 3b represent that compound **3** has higher mortality as 97.50% and 79.17% at 50 μg/mL with exposure of 72 h and 48 h by feeding assay respectively. Similarly, by contact toxicity assay 79.17% and 54.17% mortality were exerted at the same concentration and time. Moreover, significant mortality was also afforded at 25 μg/mL by feeding as well as contact assay i.e. 80.83%; 66.67% and 66.67%; 46.67% mortality at 72 h and 48 h respectively. Similar results for mortality were also displayed by compound **4**, where, 100% and 85.00% mortality were reported at 50 μg/mL at exposure of 72 h and 48 h respectively. Similarly, 92.50%, 76.66% and 66.67% mortality was displayed at 25 μg/mL, 12.5 μg/mL and 6.5 μg/mL at 72 h respectively. However, via contact assay maximum 87.50% mortality was recorded at 72 h and 50 μg/mL concentration (Fig. 3b). Results for insecticidal activity of compound **5** were presented in Fig. 3c which showed that 70.83% and 66.67% mortality were displayed at 72 h at the concentration of 50 μg/mL and 25 μg/mL respectively by feeding assay. Similarly, by contact assay 53.33% and 50.83% mortality were recorded at the same concentration and time exposure respectively. Same trend of mortality was exerted by compound **6** where 41.67% and 36.67% mortality were recorded at 50 μg/mL and time exposure of 72 h (Fig. 3c). Alternatively, compound **7** was the best compound that presented significantly high mortality i.e. 100% and 85.00% mortality via feeding assay at 50 μg/mL concentration with 72 h exposure. Similarly, at 25 μg/mL 92.50% and 71.67% mortality were recorded at the same exposure period. Interestingly, this compound displayed 66.67% mortality at 72 h exposure even at low concentration 6.5 μg/mL. Whereas, relatively lower mortality i.e. 71.67% and 65.83% were recorded by contact assay at 50 μg/mL and 25 μg/mL and 72 h exposure respectively (Fig. 3d). Results presented in Fig. 3d for compound **8** described that maximum mortality was afforded at 50 μg/mL and 25 μg/mL with 72 h and 48 h as 90.00% and 81.67% respectively by feeding toxicity assay, whereas, at the same concentration and exposure period 61.67% and 57.56% mortality were recorded by contact assay. Interestingly, compound **9** produced higher mortality on using contact assay rather than feeding assay like other described compounds. However, this compound produced moderate mortality in both bioassays i.e. 66.67%; 51.67% and 64.17%; 50.83% at 50 μg/mL at 72 h and 48 h exposure respectively (Fig. 3e). On the other hand, compound **10** recorded relatively higher mortality as 80.83% and 68.33% by contact toxicity assay at 50 μg/mL and 25 μg/mL and 72 h exposure period respectively as compared to contact toxicity assay which afforded 56.67% and 46.83% mortality at the same concentration and exposure period.

In essence, compound **3** exhibited an extraordinary mortality against *B. tabaci* on both contact and feeding experiments. Compound **4** and **7** highlighted the significant insecticidal activities on feeding bioassay and strong insecticidal activities on contact bioassay while compound **5**, **8** and **10** demonstrated their strong feeding toxicity effectiveness while their contact insecitidal activities were moderate. Comparing the values above, compounds **1**, **2**, **6** and **9** ranged from moderate to fair effectiveness to *B. tabaci.* However, the organic solvents methanol and dichloromethane used as diluting agents in the contact assay did not cause any mortality.

Probability analysis showed the LC_50_ values, slope value, Chi-square and fiducial limits at 95% confidence limit. Lowest LC_50_ values displayed by compounds **3**, **4** and **7** as 11.42, 5.66 and 5.65 µg/mL and 10.59, 19.05 and 26.59 µg/mL respectively via feeding and contact assay which illustrates their extraordinary insecticidal activity against *B. tabaci*. Whereas, other compounds displayed higher LC_50_ values via feeding as well as contact assay respectively that showed their less toxicity against said pest (Table 2).

### Safety evaluation of active compounds

It is also important to note here that no phytotoxicity was observed during the whole experiment. The data presented in Table 3 shows the negligible effects of different compounds on height of pepper, cucumber, tomato and cotton were observed. Moreover, the data of pepper and cucumber presented in the Table 3 are highly significant while the data of tomato and cotton in Table 3 are moderatly to highly significant. Similarly, no phytotoxicity on leaves or stem was observed. After treatment on the 7th and 14th days were found healthy without any abnormalities like discoloration, necrosis, growth retardation, wilting and deformity.

**Table 3:** Effects of active compounds on crop heights at 100 µg/mL.

Compound	Pepper height (cm)			Cucumber height (cm)		
	Before spray	7th Day	14th Day	Before spray	7th Day	14th Day
**3**	16.53 ± 1.0^c^	21.50 ± 0.7^b^	27.50 ± 0.6^a^	7.23 ± 0.3^c^	11.57 ± 0.6^b^	15.17 ± 0.5^a^
**4**	16.83 ± 0.3^c^	22.13 ± 1.4^b^	28.80 ± 0.7^a^	7.67 ± 0.9^c^	12.27 ± 0.4^b^	14.77 ± 0.7^a^
**5**	16.40 ± 0.5^c^	20.57 ± 0.9^b^	28.20 ± 0.8^a^	7.40 ± 0.3^c^	12.07 ± 0.3^b^	15.13 ± 0.6^a^
**7**	16.73 ± 0.7^c^	21.37 ± 0.6^b^	27.70 ± 0.6^a^	8.10 ± 0.7^c^	11.87 ± 1.1^b^	14.67 ± 0.3^a^
**8**	17.03 ± 0.6^c^	20.90 ± 0.9^b^	28.23 ± 0.6^a^	7.80 ± 0.4^c^	12.00 ± 0.6^b^	15.23 ± 0.8^a^
**9**	16.80 ± 0.6^c^	21.67 ± 1.0^b^	27.00 ± 0.4^a^	7.47 ± 0.5^b^	13.00 ± 1.0^a^	14.47 ± 0.4^a^
**10**	16.86 ± 0.8^c^	23.26 ± 0.6^b^	28.60 ± 0.4^a^	8.40 ± 0.6^c^	12.33 ± 0.7^b^	14.60 ± 0.3^a^
**CK**	17.43 ± 0.6^c^	22.86 ± 0.4^b^	27.86 ± 0.7^a^	7.27 ± 0.6^c^	11.33 ± 0.3^b^	15.00 ± 0.6^a^

Compound	Tomato height (cm)			Cotton height (cm)		
	Before spray	7th Day	14th Day	Before spray	7th Day	14th Day
**3**	10.47 ± 04.^c^	13.17 ± 0.2^b^	15.83 ± 0.6^a^	12.97 ± 0.3^b^	13.6 ± 0.4^ab^	14.60 ± 0.3^a^
**4**	10.36 ± 0.6^c^	12.80 ± 0.5^b^	15.63 ± 0.6^a^	12.53 ± 0.3^b^	13.8 ± 0.6^ab^	15.10 ± 0.3^a^
**5**	10.33 ± 0.9^b^	12.00 ± 0.2^ab^	15.20 ± 1.4^a^	12.36 ± 0.3^b^	13.40 ± 0.4^ab^	14.43 ± 0.2^a^
**7**	9.30 ± 0.6^a^	12.60 ± 0.5^a^	15.53 ± 0.7^a^	12.20 ± 0.2^c^	13.80 ± 0.2^b^	15.36 ± 0.6^a^
**8**	10.23 ± 0.5^c^	12.83 ± 0.9^b^	16.13 ± 0.8^a^	12.63 ± 0.3^c^	13.4 ± 0.3^ab^	14.37 ± 0.3^a^
**9**	10.13 ± 0.2^c^	13.23 ± 0.9^b^	16.20 ± 0.3^a^	12.87 ± 0.7^a^	13.63 ± 0.6^a^	14.40 ± 0.4^a^
**10**	10.53 ± 0.5^b^	12.70 ± 0.9^b^	15.80 ± 0.9^a^	12.13 ± 0.3^c^	13.76 ± 0.4^b^	15.13 ± 0.4^a^
**CK**	10.50 ± 0.6^c^	13.30 ± 0.7^b^	16.00 ± 0.2^a^	12.60 ± 0.3^b^	13.40 ± 0.4^b^	14.90 ± 0.3^a^

Data in the columns presented as mean values ± standard error with various superscripts are significantly different according to DMRT (P > 0.05).

## Discussion

Several problems are associated with the use of synthetic chemicals for pest management, the introduction of natural products for this purpose is the primary concern. Essential oils, extracts and biologically active compounds are commonly used due to their effectiveness and safety for the environment as well as for humans. Secondary metabolites or natural products isolated from botanical source and range of microorganisms displayed bioactivity such as insecticidal, microbial, fungicidal and cytotoxic properties and contribute to their survival in several ways^29^. However, in the current research study all isolated compounds were evaluated for their in-vitro insecticidal activity against *Bemisia tabaci*. Different compounds displayed variable insecticidal activities from excellent to moderate activity.

The toxicity results of spinasterol,22,23-dihydrospinasterol reported by Maqsood et al. are in accordance with the results that documented LC_50_ values of 32.36 and 44.49 µg/mL by contact as well as residual assay respectively against *Brevicoryne brassicae*^10^. R.M. Zolotar et al. screened out ecdysteroids and were evaluated for their insecticidal activity against the Colorado beetle (*Leptinotarsa decemlineata*) using a contact feeding method, that showed significant activity for isolated compounds^30^. The isolation of appropriate sterol from the *Beauveria* sp. and its activity against *B. tabaci* are in accordance with the findings of R.M. Zolotar et al. Similar results on insecticidal activity of ergosterol peroxide isolated from *Nomuraea rileyi* against tobacco cutworm were documented by Pannipa Prompiboon et al.^31^ who reported the moderate effects such as 46.7% mortality was caused against *Spodoptera litura* larvae via topical application at an interval of one week with 90–120 µg/insect, whereas, our research results showed 38.33% mortality at 72 h exposure at concentration of 50 μg/mL on *B. tabaci*. Additional biological activities of compounds ergosterol peroxide include antioxidant activities^32^, antimycobacterial activities^33^ inflammatory activities^34^. Furthermore, the research study did not find any published material with the same compounds that was isolated on insecticidal activity study. This suggests there is still great potential of prospects on finding new active molecules from known entomopathogenic fungi.

*B. tabaci* is a potent agricultural pest with strong tendency to develop resistance against known pesticides, therefore new controlling agents are in constant need. In this study methyl 1,4-dihydro-4-oxo-2-quinolinecarboxylate (**4**), bassiatin (**3**), 6-dehydrocerevisterol (**7**) have shown promising insecticidal activity against *B. tabaci*. Compounds **1, 2, 4–9** are discovered in *Beauveria* sp. for the first time and compound **4** was discovered in natural products for the first time. Compound **4** was previously synthesized by Mazzoni^22^, even this is a known compound, we still use 2D NMR to fix the structure, due to no ^13^C NMR data was reported in the literature. However, our findings on phytotoxicity and growth parameter/ height displayed that no phytotocxic effects on plants leaves and plant heights were observed which showed the safety profile of the isolated compounds.

Although different biological control approaches have been employed in current agriculture systems to control pest on crops and vegetables, however, the use of fungal based biological compounds against *B. tabaci* is limited. The outcomes from this current study offered that isolated compounds from *Beauveria* sp. useful for the control of sucking pest especially *B. tabaci.* Hence, the introduction of these biologically active compounds could be an effective alternative and potential means to control such pests.

## Materials and methods

### General experimental procedures

NMR spectra were captured on an Avance-600 NMR spectrometer (Bruker, 57 Karlsruhe, Germany) at room temperature. High-resolution electrospray ionization mass spectrometry (HRESIMS) spectra data were recorded on a 6500 series quadrupole-time-of-flight (Q-TOF) mass spectrometer (Agilent, Santa Clara, CA). Mass spectrometry also recorded on LCMS 8050 (Shimadzu, Tokyo, Japan). High-performance liquid chromatography (HPLC) analysis was performed on a 1260 Infinity LC system (Agilent, Santa Clara, CA), and the column used was a 250 mm × 4.6 mm i.d., 5 µm, ZORBAX Eclipse XDB (Agilent, Santa Clara, CA). Semipreparative HPLC was performed on a 1260 series system (Agilent), and the column used was a 250 mm × 9.4 mm i.d., 5 μm, ZORBAX Eclipse XDB (Agilent). Column chromatography was performed using silica gel (100−200 mesh) (Qingdao Ocean Chemical Co. Ltd., Qingdao, China) and Sephadex LH-20 (GE Healthcare, Uppsala, Sweden). All chemical reagents were purchased from a chemical reagent company (Sinopharm Chemical Reagent Co., Ltd., Shanghai, China) and used without further purification.

### Fungal material

The fungal strain was isolated from an asian corn borner (*Ostrinia furnacalis* Guenée) cadaver which was collected from Liaoyang city (41° 16′ 38.2" N, 123° 07′ 50.2" E), northeast of China in May, 2019. It was identified as *Beauveria* sp. LY2, according to morphological characters and molecular biological protocol by DNA amplification and sequencing of the ITS region^35^ (deposited in Genbank, accession no. SUB9703999 LY2 MZ262366). The fungal strain was prepared on sucrose agar with yeast extract slants and stored in Shenyang Agricultural University (SYAU), China, at 4 ℃.

### Fermentation and extraction of *Beauveria* sp. LY2

The *Beauveria* sp. LY2 was obtained from the Laboratory of Microbial Metabolites, College of Plant Protection of Shenyang Agricultural University, China, which was cultured on SDAY medium at 25 ℃ in an incubator. The fermentation has two stages where the first stage, SDY medium (1% peptone, 1% yeast extract, 4% glucose, pH = 7.2) was used. Put a 0.5 cm diameter fungi pancake with SDAY medium into a 250 mL Erlenmeyer flask which contains 40 mL of SDY medium, then incubated them at 25 ℃ with 180 rpm shaking speed for 3 days as to prepare the seed culture. In the second stage, Czapek–Dox medium (0.03% NaNO_3_, 0.01% K_2_HPO_4_·3H_2_O, 0.005% MgSO_4_·7H_2_O, 0.005% KCl, 0.0001% FeSO_4_·7H_2_O, 3% sucrose, pH = 7.0) was used. 120 2L Erlenmeyer flasks, each contains 400 mL Czapek–Dox medium, were inoculated with 40 mL seed culture at 25 ℃ with 180 rpm shaking speed for 15 days. The fermentation cultures were centrifuged at 4 ℃ with 5000 rpm for 30 min to remove mycelia, then add 3% Amberlite XAD 16 resin into the broth at 25 ℃ with 180 rpm shaking speed for 4 h. Resin was collected by using 100 meshes gauze and extracted four times with methanol. The dried crude extract was harvested through reduced pressure concentration. The dried methanol extract was dissolved in 600 mL solution (50% CH_3_OH, 50% H_2_O), the solution was extracted four times by 600 mL CH_2_Cl_2_. Collecting the resulted extract CH_2_Cl_2_ solution then concentrate it to produce 10.0 g solid brown residue.

### Isolation and purification

The concentrated extract was purified by using silica gel chromatography to elute stepwise with CH_2_Cl_2_-MeOH (100:0, 50:1, 25:1, 10:1 and 0:100, 1.5 L each) as the mobile phase to afford six fractions, A to G. C was separated via silica gel chromatography with PE:EA (9:1, 4:1, 7:3, 6:4 and 1:1, 300 mL each ) as the mobile phase to yield compound **3** (110 mg), compound **9 **(7.3 mg) and compound **10** (14.4 mg). Fraction E was subjected to gel chromatography on Sephadex LH-20 eluted with CH_2_Cl_2_-MeOH (1:1) to obtain compound **5** (16.7 mg). D, F, G was purified by reverse-phase semi-preparative HPLC applying a MeOH-H_2_O gradient (contain 0.1% HCOOH) respectively. Compound **6** (2 mg) was gained from D with 90% MeOH on 40 min (see Figure S26), compound **7** (4 mg) and compound **8** (4.2 mg) were also obtained from D with 85% MeOH on 18 min and 20 min (see Figures S30 and S34). Compound **4** (3.1 mg) was yielded from F with 77% MeOH on 28 min (see Figure S19), **1** (3.8 mg) and **2** (25.9 mg) was obtained from G with conditions with 98% MeOH on 10 min and 16 min respectively (see Figures S7 and S12).

### Evaluation of insecticidal activity against *B. tabaci*

#### *Bemisia tabaci* culture

The whiteflies B-biotype *B. tabaci* (MEAM1) are from Liaoning Key Laboratory of Economic and Applied Entomology was cultured in a controlled greenhouse at 25-27 ℃, 60%-70% relative humidity (RH) with 16:8 (light: dark) photoperiod, on around 2 months old cotton plants, *Gossypium hirsutum*.

#### Pre-experiment container preparation

One side of a lightproof bi-pass glass tube (3 cm inside diameter and 6 cm height) was covered with a stretched Parafilm M membrane (the first layer). Another side sealed with one piece of 100 meshes gauze and a rubber band^36^. All items had to be completely sterile.

#### Experiment of feeding insecticidal activity to *B. tabaci*

The whitefly diet comprised of 30% (w/v) sucrose with serial dilutions of each compound (50, 25, 12.5, 6.25 μg/mL respectively), 2.5 μg /mL acetamiprid was used as positive control, each diet contained 0.5% Tween 80 and 0.002% DMSO. Diet aliquots (200 μL) containing compounds were dispensed on top of the first layer of stretched Parafilm membrane over each container; another stretched Parafilm membrane was placed over the diet aliquots to prevent evaporation. 30 adults (2–5 d old) were aspirated from *G. hirsutum* into the container. Test containers were placed in a controlled greenhouse at 25–27 ℃, 60–70% under 16:8 (light:dark) photoperiod and mortality was recorded at 24, 48 and 72 h. Each iteration of the experiment consisted of four replicates for each concentration of each compound (4 replicates × 4 concentrations × 3 repetitions × 30 whiteflies per tube = 1440 *B. tabaci* per test compound).

#### Experiment of contact insecticidal activity to *B. tabaci*

Used mixed solvent (methanol: dichloromethane = 1:1) to diluent each compound into serial concentrations (50, 25, 12.5, 6.25 μg/mL respectively), used 2.5 μg/mL acetamiprid as positive control, mixed solvent with compounds aliquots were dispensed into the container tube, rotated the tube once to let the solvent equally distributed and evaporate. 30 adults (2–5 d old) were aspirated from *G. hirsutum* into the container. Test containers were placed in the controlled greenhouse at 25–27 ℃, 60–70% under 16:8 (light: dark) photoperiod. *B. tabaci* mortality was recorded at 24, 48 and 72 h. Each iteration of the experiment consisted of four replicates for each concentration of the each compound (4 replicates × 4 concentrations × 3 repetitions × 30 individual per tube = 1440 *B. tabaci* per test compound).

### Safety evaluation of active compounds

pepper (*Capsicum annuum* L., cultivar ‘Wanhao A5’ from SYAU, China), cucumber (*Cucumis sativus* L., cultivar ‘Cuibao’ from SYAU, China), Tomato (*Lycopersicon esculentum*, cultivar ‘Dafen’ from SYAU, China) and cotton (*Gossypium hirsutum*, cultivar ‘Guoshenhan 284’ from Xingyuan Co., China) were chosen as experimental objects and planted in nutrition pots from seeds for one month until they reached to specific leaves stage (tomato and pepper for 8–12 leaves, cucumber and cotton for 4–5 leaves) in greenhouse with the temperature scale between 10 and 35 ℃. The equivalent crop plants with similar heights and proper leave sizes were picked and transplanted into 7 cm^3^ black plastic pots, then divided the crop plants into 8 groups, each group consists of 3 pots for each kind of crop plants.

The insecticidal compounds **3**–**5**, **7**–**10** were diluted with water containing 0.5% DMSO and 0.5% Tween 80 into the concentration of 100 μg/mL solutions respectively. A hand-held vacuum sprayer was used to foliar spray each group of plants with 15 mL of solution per group. Plants were monitored for heights and abnormalities (discoloration, necrosis, growth retardation, wilting, deformity) every 7 days for 2 weeks according to Chinese National Standard of Laboratory Test For Crop Safety Evaluation^37^ and data was calculated.

All the studies on plants totally complied with relevant institutional, national, and international guidelines and legislation.

### Statistical analysis

All the calculated data on mortality and plant heights were analyzed via one way analysis of variance (ANOVA), the mean difference between treatments was envisioned for significance test by Duncan multiple range test DMRT with IBM-SPSS statistics 25.0 version software. Probit analysis was performed using EPA Probit analysis program version 1.5. 0.

### Figure software

All the chemical structures including bold bonds and arrows were drawn with ChemDraw 19.0. The Fig. 3 was drawn with Origin 2019b (9.65).

## Conclusions

The present research study indicated that the secondary metabolites from *Beauveria* sp. possess potential botanical agents. Results also demonstrated that *B. tabaci* produced significant sensitivity to isolated bassiatin (**3**) via both feeding and contact bioassay as well as methyl 1,4-dihydro-4-oxo-2-quinolinecarboxylate (**4**) and 6-dehydrocerevisterol (**7**) via feeding bioassay. cerevisterol (**5**), (22*E*,24*R*)-ergosta-8(14),22-diene-3β,5α,6β,7α-tetrol (**8**), melithasterol B (**9**) and ergosterol peroxide (**10**) displayed moderate toxicity against this pest. In contrast, cerebrosides F (**1**) and cerebrosides B (**2**) showed much lower mortality than other compounds. Moreover, all the compounds were found safe without affecting the plants growth with no phytotoxicity. Therefore, except compounds **1** and **2**, these compounds were introduced as alternatives to synthetic chemical insecticides. However, more research on the purification and characterization of bioactive compounds from entomopathogenic fungi is needed to be compared against *B. tabaci* and other agricultural insect pests in potential future research studies.

## Supplementary Information

Supplementary Information.
