# Supplementary material for: Isolation, purification and identification of biological compounds from Beauveria sp. and their evaluation as insecticidal effectiveness against Bemisia tabaci
Source: Sci Rep. 2021 Jun 8;11:12020. doi: 10.1038/s41598-021-91574-9 (PMC8187347; doi:10.1038/s41598-021-91574-9)
Supplement: Supplementary file 1 — Supplementary Information. [file 41598_2021_91574_MOESM1_ESM.docx]

**Supplementary Material**

Isolation, purification and identification of biological compounds from *Beauveria* sp. and their evaluation as insecticidal effectiveness against *Bemisia tabaci*

**Ran An ^1^, Maqsood Ahmed ^1, 2^ , Haiyan Li ^1^, Yanbin Wang ^1^ , Aimin Zhang ^1^ , Yuhui Bi ^1^ and Zhiguo Yu ^1,^***

**^1^** College of Plant Protection, Shenyang Agricultural University , Shenyang 110866, China;

[anan8u8@outlook.com](mailto:anan8u8@outlook.com) (R.A.); [maqsoodahmed200@hotmail.com](mailto:maqsoodahmed200@hotmail.com) (M.A.); [lihaiyan98@163.com](mailto:lihaiyan98@163.com) (H.L.); [ybwang_yqh@163.com](mailto:ybwang_yqh@163.com) (Y.W.); [zhangaimin99@126.com](mailto:zhangaimin99@126.com) (A.Z.); [biyuhui@syau.edu.cn](mailto:biyuhui@syau.edu.cn) (Y.B.)

^2^ Department of Agriculture, Pest Warning & Quality Control of Pesticides, Gujrat 50700, Pakistan

* Correspondence: [zyu@syau.edu.cn](mailto:zyu@syau.edu.cn) (Z.Y.); Tel.: +86-24-8848-7148 (Z.Y.)

**Contents**

[Figure S1. ^1H^ NMR spectrum (MeOD, 600 MHz) of cerebroside F (1) 6](#_Toc72540596)

[Figure S2. ^13C^ NMR spectrum (MeOD, 600 MHz) of cerebroside F (1) 7](#_Toc72540597)

[Figure S3. ^1H 13C^ HMBC NMR spectrum (MeOD, 600 MHz) of cerebroside F (1) 8](#_Toc72540598)

[Figure S4. ^1H 13C^ HSQC NMR spectrum (MeOD, 600 MHz) of cerebroside F (1) 9](#_Toc72540599)

[Figure S5. ^1H 1H^ COSY NMR spectrum (MeOD, 600 MHz) of cerebroside F (1) 10](#_Toc72540600)

[Figure S6. HR-ESI-MS data of cerebroside F (1) 11](#_Toc72540601)

[Figure S7. HPLC signal of cerebroside F (1) 11](#_Toc72540602)

[Figure S8. HR-ESI-MS fragments data of cerebroside F (1) 15](#_Toc72540603)

[Table S1: Insecticidal activity of compound 1 16](#_Toc72540604)

[Figure S9. ^1H^ NMR spectrum (DMSO, 600 MHz) of cerebroside B (2) 17](#_Toc72540605)

[Figure S10. ^13C^ NMR spectrum (DMSO, 600 MHz) of cerebroside B (2) 18](#_Toc72540606)

[Figure S11. HR-ESI-MS data of cerebroside B (2) 19](#_Toc72540607)

[Figure S12. HPLC signal of cerebroside B (2) 19](#_Toc72540608)

[Table S2. Insecticidal activity of compound 2 20](#_Toc72540609)

[Figure S13. ^1H^ NMR spectrum (CDCl_3_, 600 MHz) of bassiatin (3) 21](#_Toc72540610)

[Figure S14. ^13C^ NMR spectrum (CDCl_3_, 600 MHz) of bassiatin (3) 22](#_Toc72540611)

[Figure S15 ESI-MS data of bassiatin (3) 23](#_Toc72540612)

[Table S3. Insecticidal activity of compound 3 24](#_Toc72540613)

[Figure S16. ^1H^ NMR spectrum (CDCl_3_, 600 MHz) of methyl 1,4-dihydro-4-oxo-2-quinolinecarboxylate (4) 25](#_Toc72540614)

[Figure S17. ^13C^ NMR spectrum (CDCl_3_, 600 MHz) of methyl 1,4-dihydro-4-oxo-2-quinolinecarboxylate (4) 26](#_Toc72540615)

[Figure S18. ESI-MS data of methyl 1,4-dihydro-4-oxo-2-quinolinecarboxylate (4) 27](#_Toc72540616)

[Figure S19. HPLC signal of methyl 1,4-dihydro-4-oxo-2-quinolinecarboxylate (4) 27](#_Toc72540617)

[Table S4. Insecticidal activity of compound 4 28](#_Toc72540618)

[Figure S20. ^1H^ NMR spectrum (CDCl_3_, 600 MHz) of cerevisterol (5) 29](#_Toc72540619)

[Figure S21. ^13C^ NMR spectrum (CDCl_3_, 600 MHz) of cerevisterol (5) 30](#_Toc72540620)

[Figure S22. ESI-MS data of cerevisterol (5) 31](#_Toc72540621)

[Table S5. Insecticidal activity of compound 5 32](#_Toc72540622)

[Figure S23. ^1H^ NMR spectrum (MeOD, 600 MHz) of 9-hydroxycerevisterol (6) 33](#_Toc72540623)

[Figure S24. ^13C^ NMR spectrum (MeOD, 600 MHz) of 9-hydroxycerevisterol (6) 34](#_Toc72540624)

[Figure S25. HR-ESI-MS data of 9-hydroxycerevisterol (6) 35](#_Toc72540625)

[Figure S26. HPLC signal of 9-hydroxycerevisterol (6) 35](#_Toc72540626)

[Table S6. Insecticidal activity of compound 6 36](#_Toc72540627)

[Figure S27. ^1H^ NMR spectrum (CDCl_3_, 600 MHz) of 6-dehydrocerevisterol (7) 37](#_Toc72540628)

[Figure S28. ^13C^ NMR spectrum (CDCl_3_, 600 MHz) of 6-dehydrocerevisterol (7) 38](#_Toc72540629)

[Figure S29. ESI-MS data of 6-dehydrocerevisterol (7) 39](#_Toc72540630)

[Figure S30. HPLC signal of 6-dehydrocerevisterol (7) 40](#_Toc72540631)

[Table S7: Insecticidal activity of compound 7 41](#_Toc72540632)

[Figure S31. ^1H^ NMR spectrum (CDCl_3_, 600 MHz) of (22E,24R)-ergosta-8(14),22-diene-3β,5α,6β,7α-tetrol (8) 42](#_Toc72540633)

[Figure S32. ^13C^ NMR spectrum (CDCl_3_, 600 MHz) of (22E,24R)-ergosta-8(14),22-diene-3β,5α,6β,7α-tetrol (8) 43](#_Toc72540634)

[Figure S33. ESI-MS data of (22E,24R)-ergosta-8(14),22-diene-3β,5α,6β,7α-tetrol (8) 44](#_Toc72540635)

[Figure S34. HPLC signal of (22E,24R)-ergosta-8(14),22-diene-3β,5α,6β,7α-tetrol (8) 45](#_Toc72540636)

[Table S8. Insecticidal activity of compound 8 46](#_Toc72540637)

[Figure S35. ^1H^ NMR spectrum (CDCl_3_, 600 MHz) of melithasterol B (9) 47](#_Toc72540638)

[Figure S36. ^13C^ NMR spectrum (CDCl_3_, 600 MHz) of melithasterol B (9) 48](#_Toc72540639)

[Figure S37. ESI-MS data of melithasterol B (9) 49](#_Toc72540640)

[Table S9. Insecticidal activity of compound 9 50](#_Toc72540641)

[Figure S38. ^1H^ NMR spectrum (CDCl_3_, 600 MHz) of ergosterol peroxide (10) 51](#_Toc72540642)

[Figure S39. ^13C^ NMR spectrum (CDCl_3_, 600 MHz) of ergosterol peroxide (10) 52](#_Toc72540643)

[Figure S40. ESI-MS data of ergosterol peroxide (10) 53](#_Toc72540644)

[Table S10: Insecticidal activity of compound 10 54](#_Toc72540645)

[Figure S41. Effects of active compounds on cotton at 100 µg/mL 55](#_Toc72540646)

[Table S11. Effects of active compounds on cotton height at 100 µg/mL 56](#_Toc72540647)

[Figure S42. Effects of active compounds on cucumber at 100 µg/mL 57](#_Toc72540648)

[Table S12. Effects of active compounds on cucumber height at 100 µg/mL 58](#_Toc72540649)

[Figure S43. Effects of active compounds on pepper at 100 µg/mL 59](#_Toc72540650)

[Table S13. Effects of active compounds on pepper height at 100 µg/mL 60](#_Toc72540651)

[Figure S44. Effects of active compounds on tomato at 100 µg/mL 61](#_Toc72540652)

[Table S14. Effects of active compounds on tomato height at 100 µg/mL 62](#_Toc72540653)

[Figure S45. The conidia of *Beauveria* sp. 63](#_Toc72540654)


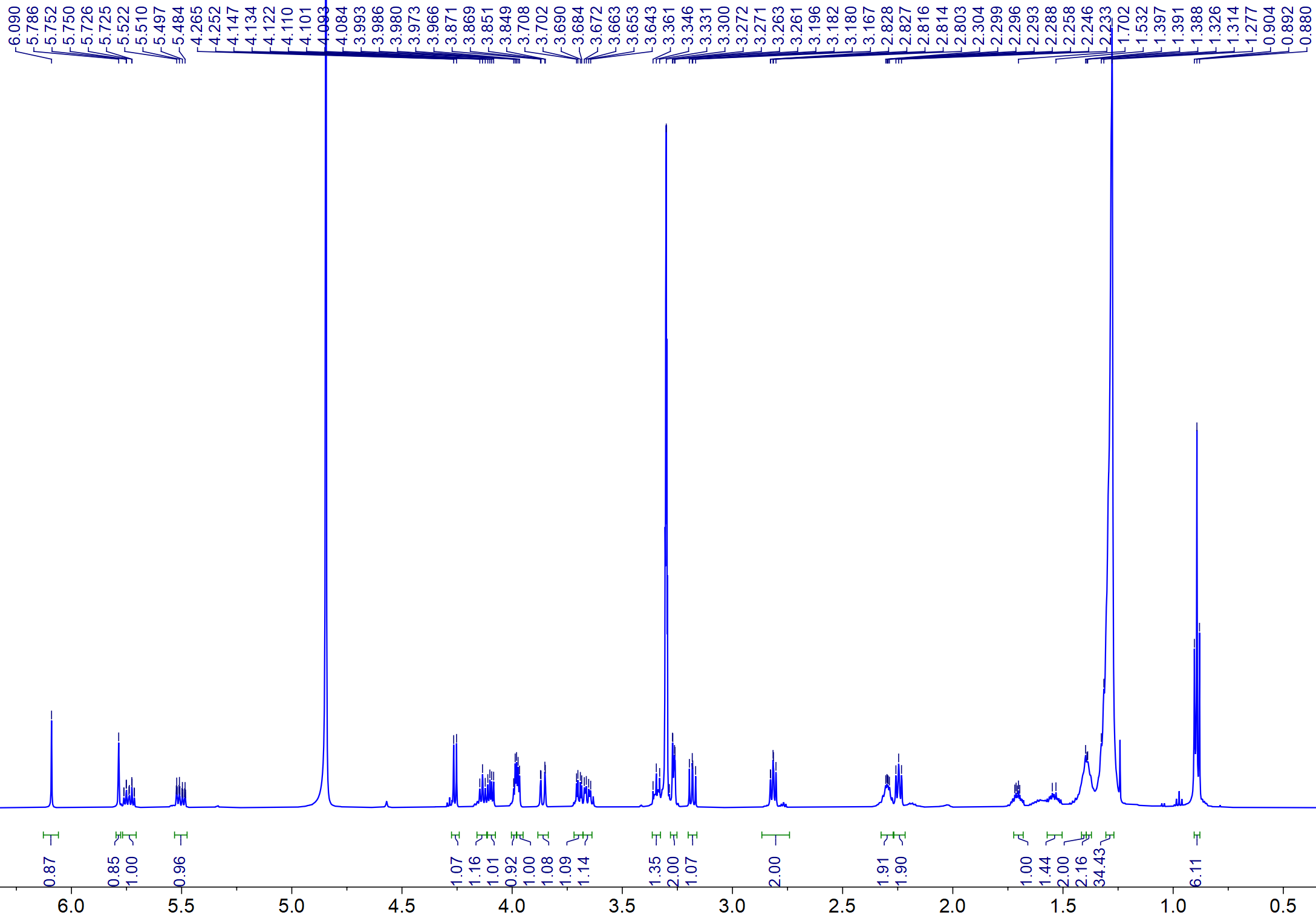


Figure S1. ^1^H NMR spectrum (MeOD, 600 MHz) of cerebroside F (**1**)


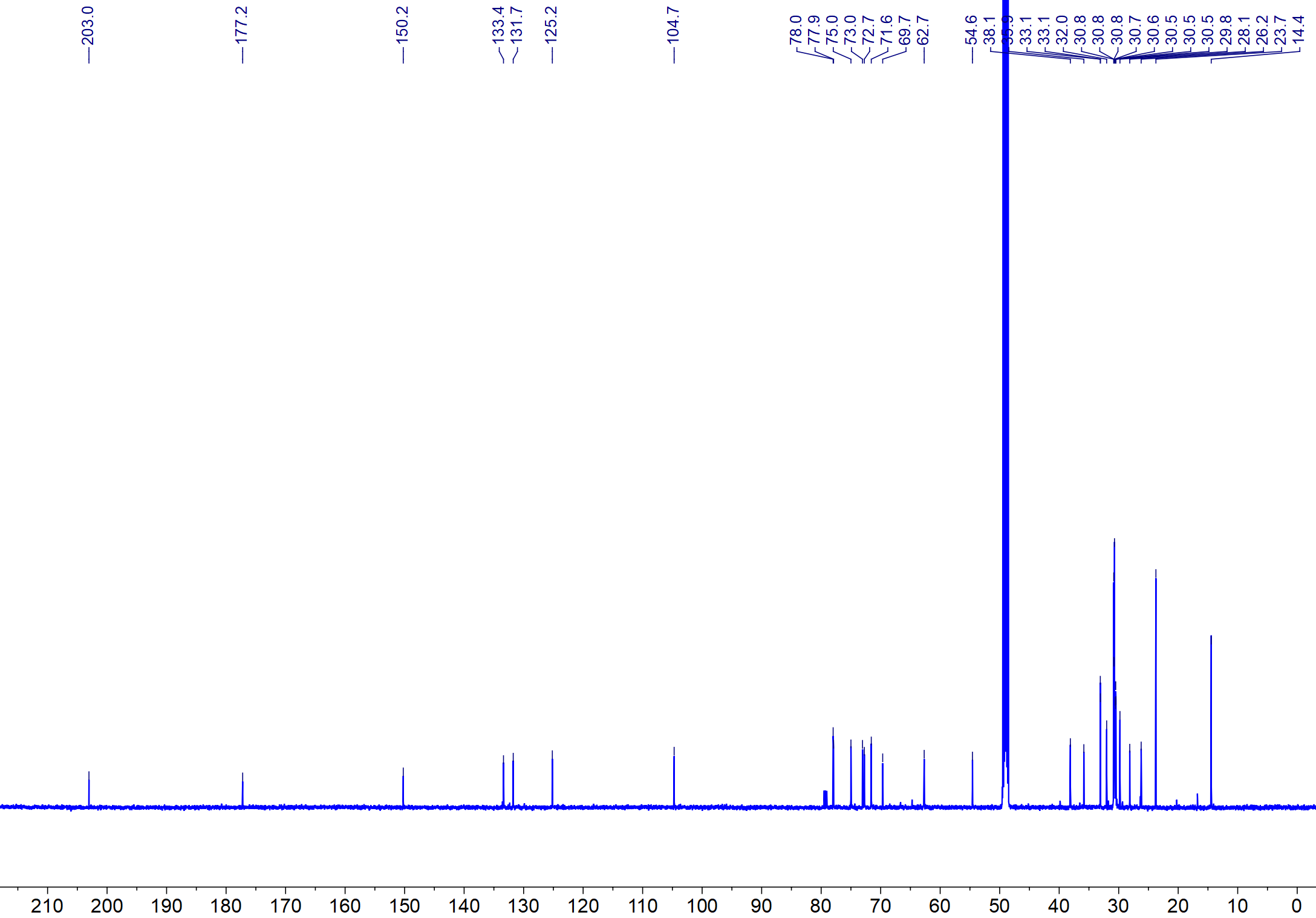


Figure S2. ^13^C NMR spectrum (MeOD, 600 MHz) of cerebroside F (**1**)


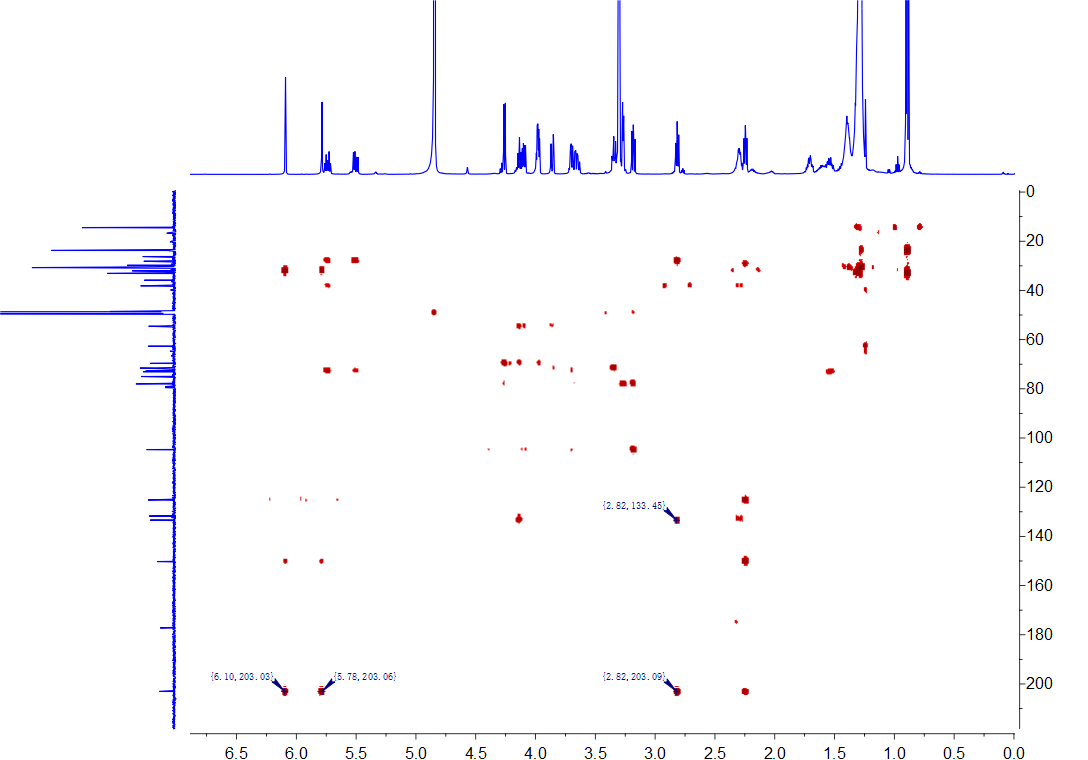


Figure S3. ^1^H ^13^C HMBC NMR spectrum (MeOD, 600 MHz) of cerebroside F (**1**)

**
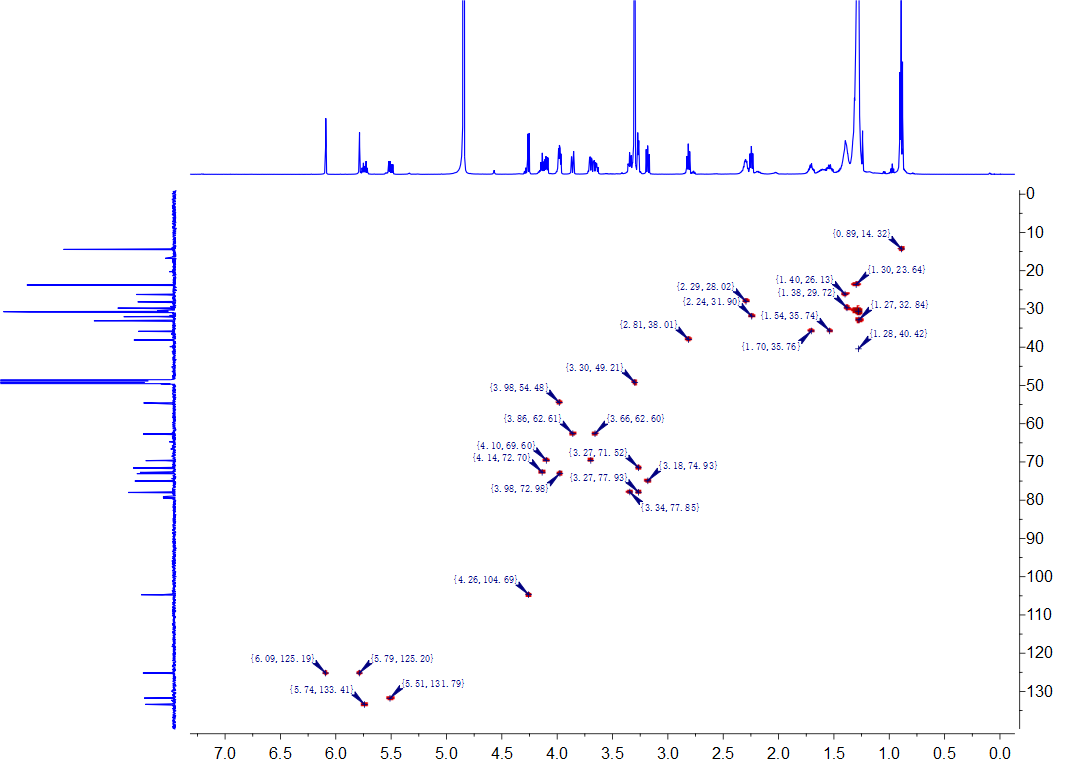
**

**Figure S4**. ^1^H ^13^C HSQC NMR spectrum (MeOD, 600 MHz) of cerebroside F (**1**)

**
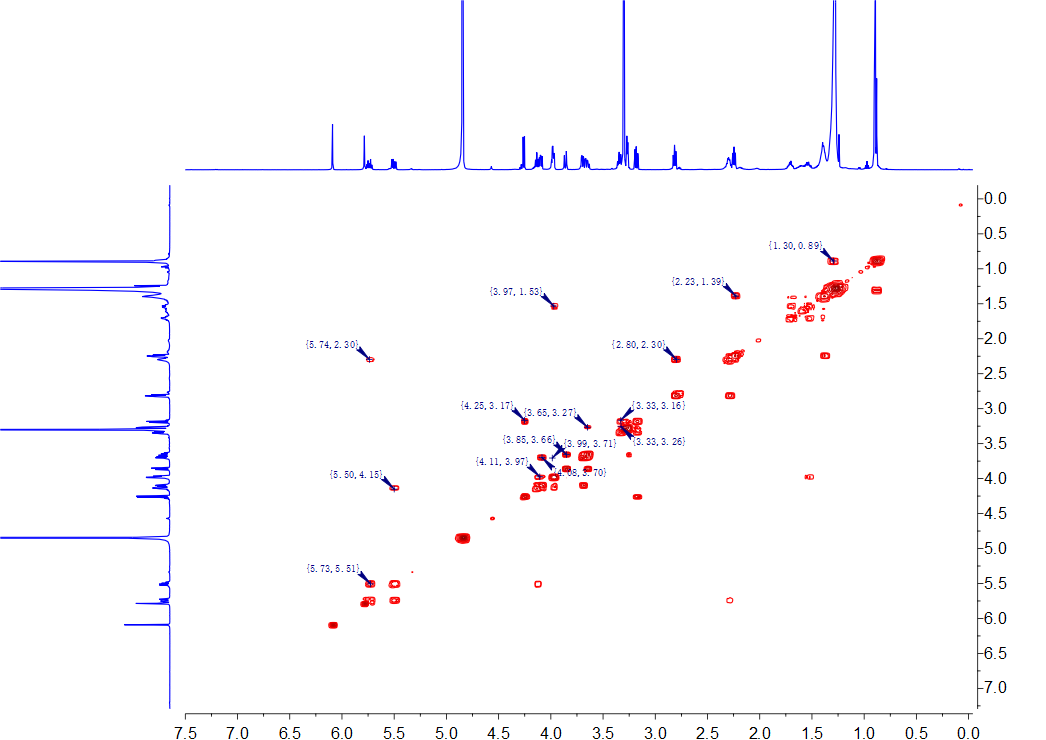
**

**Figure S5**. ^1^H ^1^H COSY NMR spectrum (MeOD, 600 MHz) of cerebroside F (**1**)

**Figure S6**. HR-ESI-MS data of cerebroside F (**1**)

**
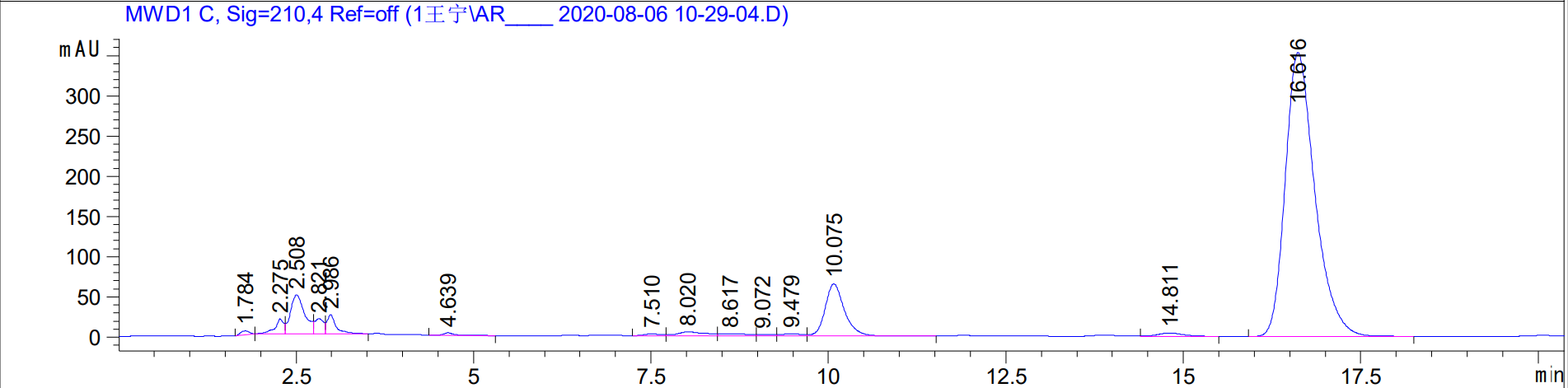
**

**Figure S7**. HPLC signal of cerebroside F (**1**)

**MS**

**MS^2^**

**Figure S8**. HR-ESI-MS fragments data of cerebroside F (**1**)

**Table S1:** Insecticidal activity of compound **1**

| **Conc. (ppm)** | **Mean mortality (%) with exposure time (h)** | | | | | |
| --- | --- | --- | --- | --- | --- | --- |
|  | **Contact toxicity assay** | | | **Feeding toxicity assay** | | |
|  | **24** | **48** | **72** | **24** | **48** | **72** |
| 50 | 13.33±6.67^b^ | 25.00±12.62^b^ | 41.67±11.05^b^ | 15.00±7.94^b^ | 22.50±13.7^b^ | 38.33±10.00^b^ |
| 25 | 6.67±2.72^b^ | 10.83±4.19^bc^ | 25.83±6.84^b^ | 16.66±11.85^b^ | 15.83±9.56^b^ | 32.49±13.71^bc^ |
| 12.5 | 5.83±5.00^b^ | 10.83±3.19^bc^ | 22.5±3.19^c^ | 10.83±11.98^b^ | 15.00±11.05^b^ | 30.83±10.67^bc^ |
| 6.25 | 4.17±4.19^b^ | 8.33±6.38^c^ | 19.17±7.83^c^ | 7.50±6.87^b^ | 7.50±6.87^b^ | 22.50±8.76^bc^ |
| PC | 80.83±5.69^a^ | 97.50±3.19^a^ | 100.00±0.00^a^ | 88.33±4.30^a^ | 100.0±0.00^a^ | 100.0±0.00^a^ |
| CK | 3.33±2.72^b^ | 5.83±1.67^c^ | 13.33±0.00^c^ | 5.00±1.92^b^ | 8.33±1.92^b^ | 15.83±1.67^c^ |
| Statics summary | |  |  |  |  |  |
| S.S | 18585 | 25164 | 20816 | 20319 | 25351 | 18533 |
| M.S | 3717.12 | 5032.9 | 4163.1 | 4063.9 | 5070.3 | 3706.4 |
| df | 5 | 5 | 5 | 5 | 5 | 5 |
| *f* | 166.1*** | 125.5*** | 113.79*** | 58.5*** | 66.11*** | 46.18*** |

Data in the columns is described as mean values ± standard deviation with various superscripts is significantly

different according to DMRT ( P> 0.05). S.S (Sum of square); Df (Degree of freedom); M.S (Mean square); F

(Significance); PC (Postive Control); CK (Check); *** (level of significance).


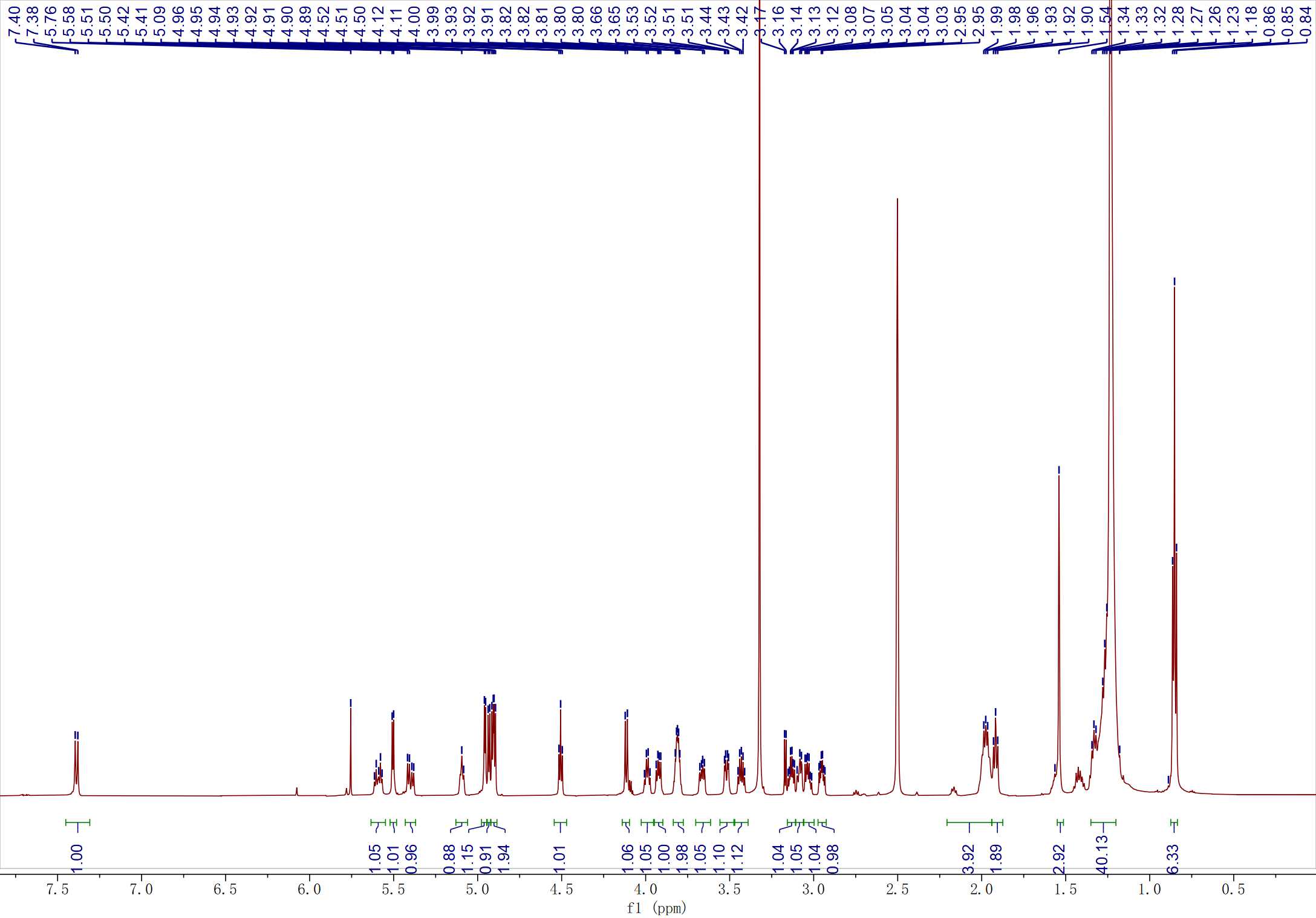


Figure S9. ^1^H NMR spectrum (DMSO, 600 MHz) of cerebroside B (**2**)


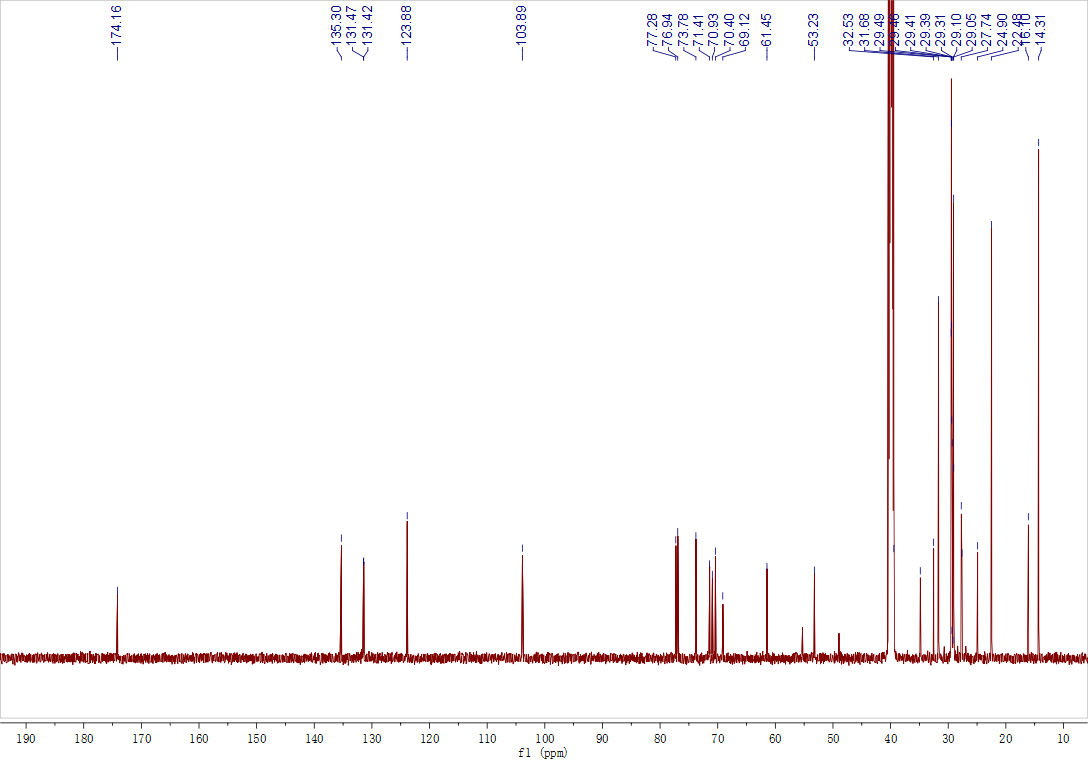


Figure S10. ^13^C NMR spectrum (DMSO, 600 MHz) of cerebroside B (**2**)

**Figure S11**. HR-ESI-MS data of cerebroside B (**2**)

**
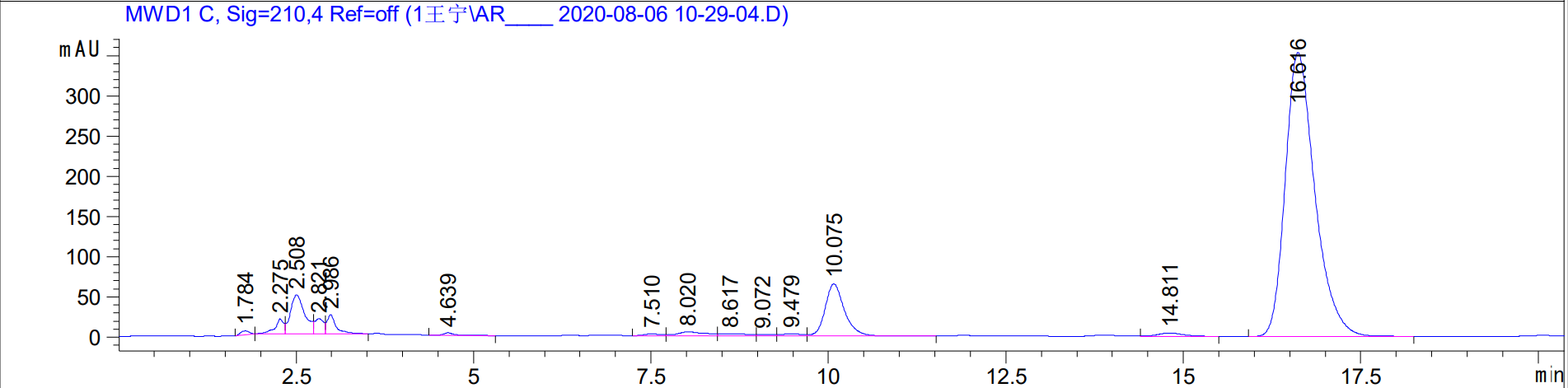
**

**Figure S12**. HPLC signal of cerebroside B (**2**)

**Table S2.** Insecticidal activity of compound **2**

| **Conc. (ppm)** | **Mean mortality (%) with exposure time (h)** | | | | | |
| --- | --- | --- | --- | --- | --- | --- |
|  | **Contact toxicity assay** | | | **Feeding toxicity assay** | | |
|  | **24** | **48** | **72** | **24** | **48** | **72** |
| **50** | 20.83±1.67^b^ | 31.66±1.92^b^ | 44.17±3.19^b^ | 15.83±12.87^b^ | 19.99±14.14^b^ | 47.50±13.43^b^ |
| **25** | 15.83±6.31^b^ | 20.00±3.84^c^ | 30.00±9.81^c^ | 9.17±5.00^b^ | 18.33±4.30^b^ | 40.83±13.97^bc^ |
| **12.5** | 5.83±5.00^c^ | 13.33±3.85^cd^ | 21.67±4.30^cd^ | 7.50±3.19^b^ | 14.16±3.19^b^ | 24.16±5.69^cd^ |
| **6.25** | 4.16±3.19^c^ | 11.67±4.30^de^ | 15.83±5.00^d^ | 6.67±6.08^b^ | 13.33±6.08^b^ | 18.33±6.38^d^ |
| **PC** | 80.83±5.69^a^ | 97.50±3.19^a^ | 100.00±0.00^a^ | 88.33±4.30^a^ | 100.0±0.00^a^ | 100.0±0.00^a^ |
| **CK** | 3.33±2.72^c^ | 5.83±1.67^e^ | 13.33±0.00^d^ | 5.00±2.72^b^ | 8.33±1.92^b^ | 15.83±1.67^d^ |
| **Statics summary** | |  |  |  |  |  |
| **S.S** | 17713 | 23427 | 21245 | 21348 | 24513 | 19814 |
| **M.S** | 3542.6 | 4685.4 | 4269.03 | 4269.6 | 4902.7 | 3962.9 |
| **df** | 5 | 5 | 5 | 5 | 5 | 5 |
| **f** | 180.72*** | 433.7*** | 169.91*** | 98.5*** | 109.2*** | 52.6*** |

Data in the columns is described as mean values ± standard deviation with various superscripts is significantly

different according to DMRT ( P> 0.05). S.S (Sum of square); Df (Degree of freedom); M.S (Mean square); F

(Significance); PC (Postive Control); CK (Check); *** (level of significance).


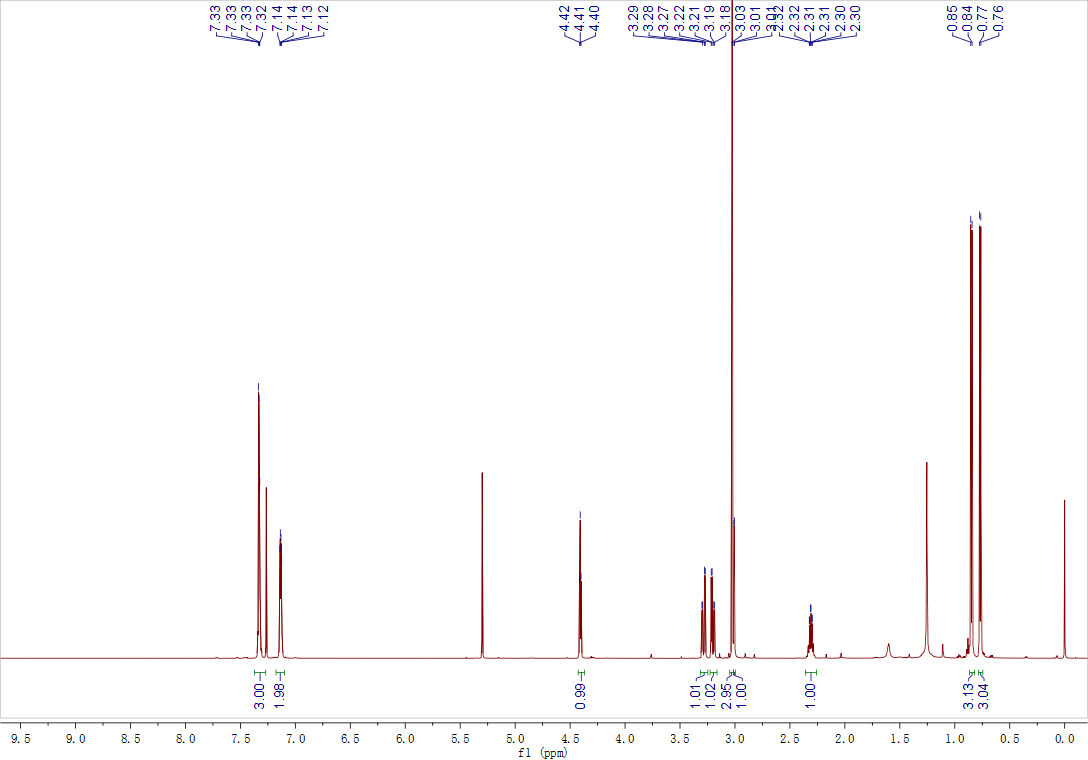


Figure S13. ^1^H NMR spectrum (CDCl3, 600 MHz) of bassiatin (**3**)


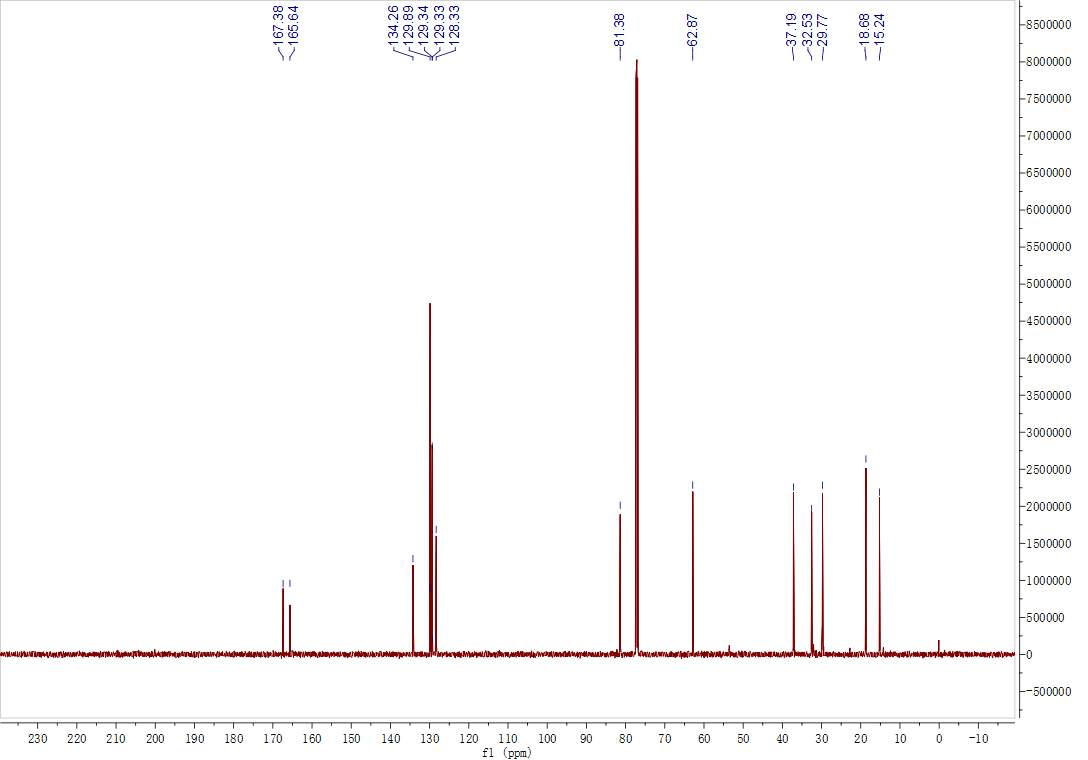


Figure S14. ^13^C NMR spectrum (CDCl3, 600 MHz) of bassiatin (**3**)


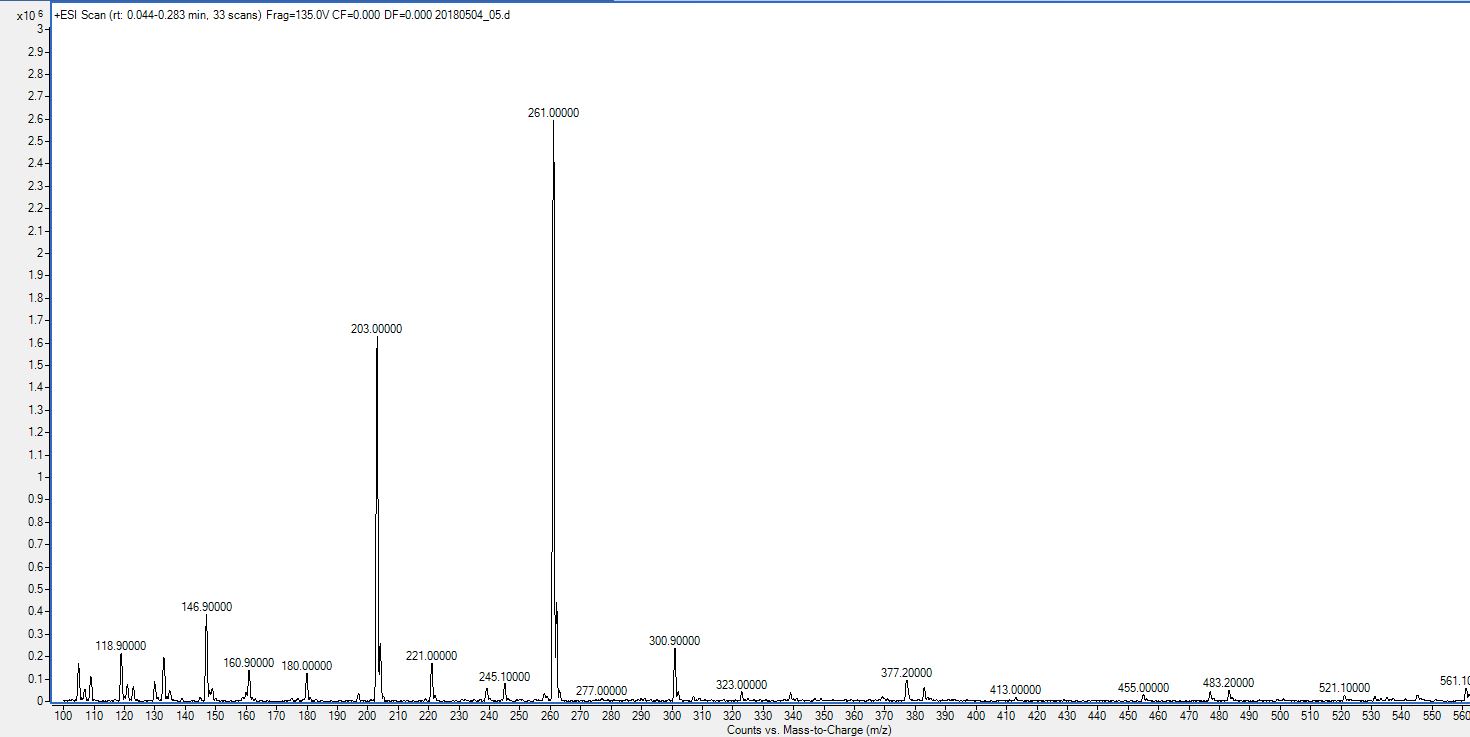


**Figure S15** ESI-MS data of bassiatin (3)

**Table S3.** Insecticidal activity of compound **3**

| **Conc. (ppm)** | **Mean mortality (%) with exposure time (h)** | | | | | |
| --- | --- | --- | --- | --- | --- | --- |
|  | **Contact toxicity assay** | | | **Feeding toxicity assay** | | |
|  | **24** | **48** | **72** | **24** | **48** | **72** |
| **50** | 31.67±4.30^b^ | 54.17±7.39^b^ | 79.17±14.24^b^ | 46.67±5.44^b^ | 79.17±6.31^b^ | 97.50±3.19^a^ |
| **25** | 23.33±0.00^bc^ | 46.67±2.72^bc^ | 66.67±7.20^bc^ | 45.00±6.94^b^ | 56.67±11.86^c^ | 80.83±9.95^b^ |
| **12.5** | 18.33±4.30^c^ | 40.00±4.71^cd^ | 68.33±5.77^bc^ | 28.33±4.30^c^ | 33.33±2.72^d^ | 62.50±7.39^c^ |
| **6.25** | 9.17±3.19^d^ | 35.00±6.93^d^ | 57.49±11.6^c^ | 25.00±4.30^c^ | 29.17±1.67^d^ | 51.67±8.82^c^ |
| **PC** | 80.83±5.69^a^ | 97.50±3.19^a^ | 100.00±0.00^a^ | 88.33±4.30^a^ | 100.0±0.00^a^ | 100.0±0.00^a^ |
| **CK** | 3.33±2.72^d^ | 5.84±1.67^d^ | 13.33±0.00^d^ | 5.00±1.92^d^ | 8.33±1.92^e^ | 15.83±1.67^d^ |
| **Statics summary** | |  |  |  |  |  |
| **S.S** | 15531 | 179851 | 16445.0 | 15964 | 23341 | 20308 |
| **M.S** | 3106.3 | 3590.3 | 3329.1 | 3192.8 | 4668.3 | 4461.7 |
| **df** | 5 | 5 | 5 | 5 | 5 | 5 |
| **f** | 214.1*** | 148.2*** | 47.1*** | 139.8*** | 144.1*** | 99.7*** |

Data in the columns is described as mean values ± standard deviation with various superscripts is significantly

different according to DMRT ( P> 0.05). S.S (Sum of square); Df (Degree of freedom); M.S (Mean square); F

(Significance); PC (Postive Control); CK (Check); *** (level of significance).


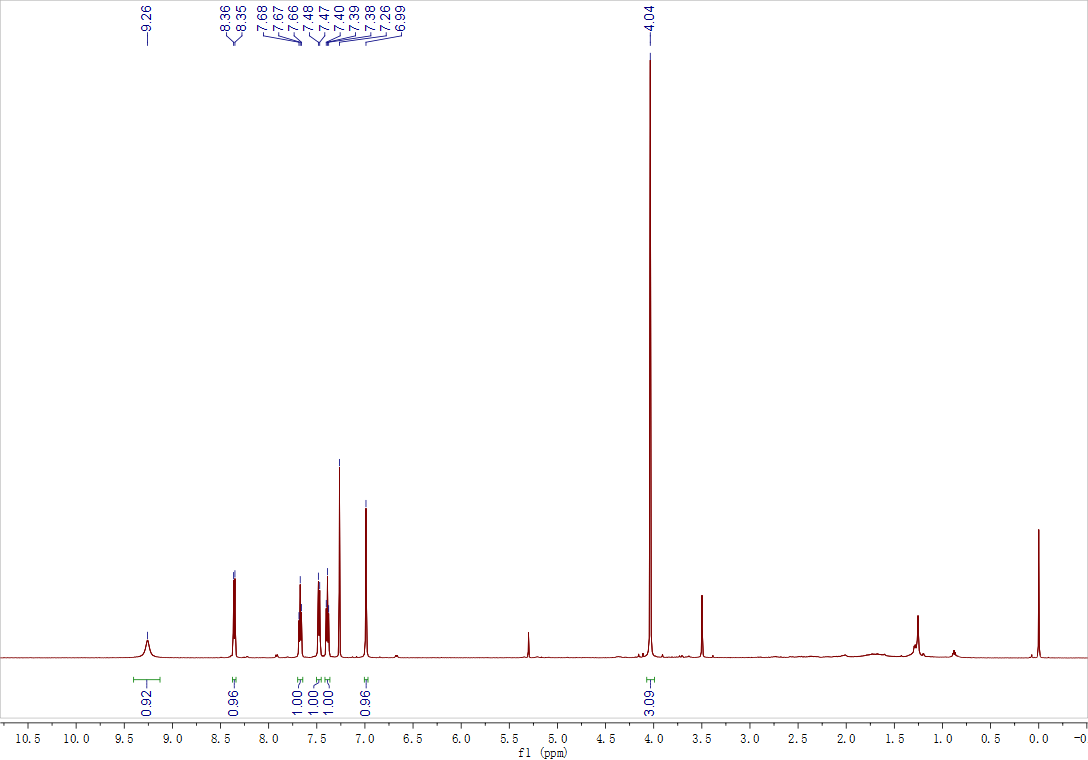


Figure S16. ^1^H NMR spectrum (CDCl3, 600 MHz) of methyl 1,4-dihydro-4-oxo-2-quinolinecarboxylate (**4**)


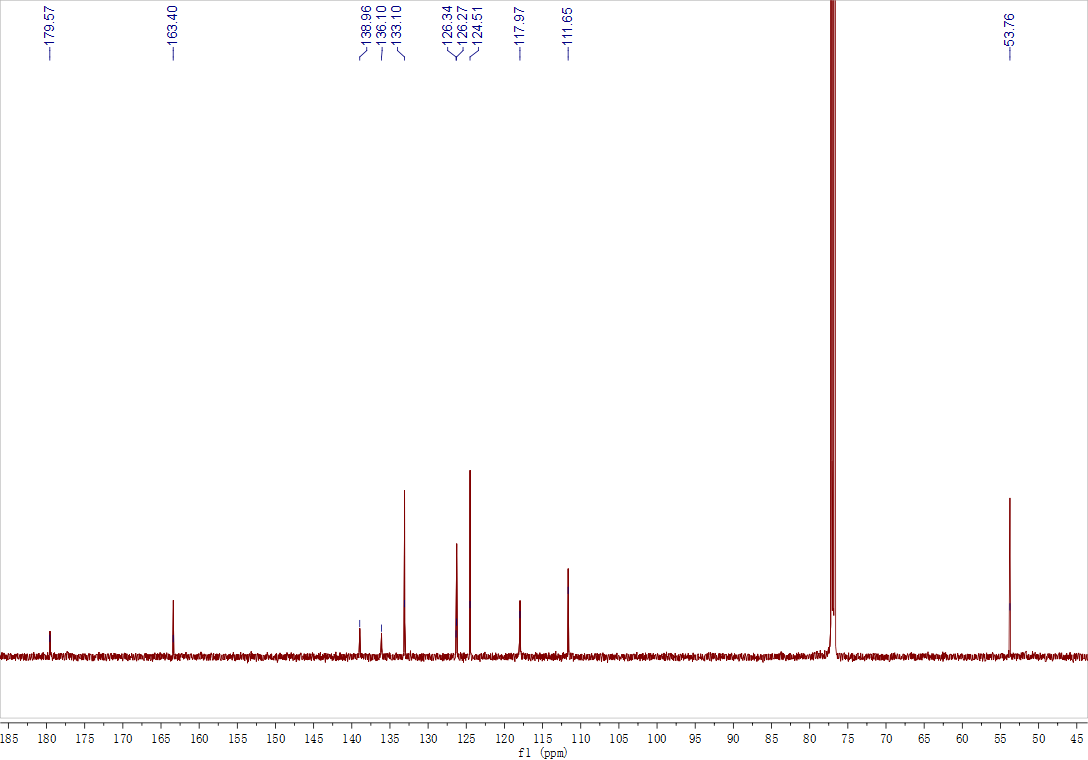


Figure S17. ^13^C NMR spectrum (CDCl3, 600 MHz) of methyl 1,4-dihydro-4-oxo-2-quinolinecarboxylate (**4**)

**Figure S18**. ESI-MS data of methyl 1,4-dihydro-4-oxo-2-quinolinecarboxylate (**4**)


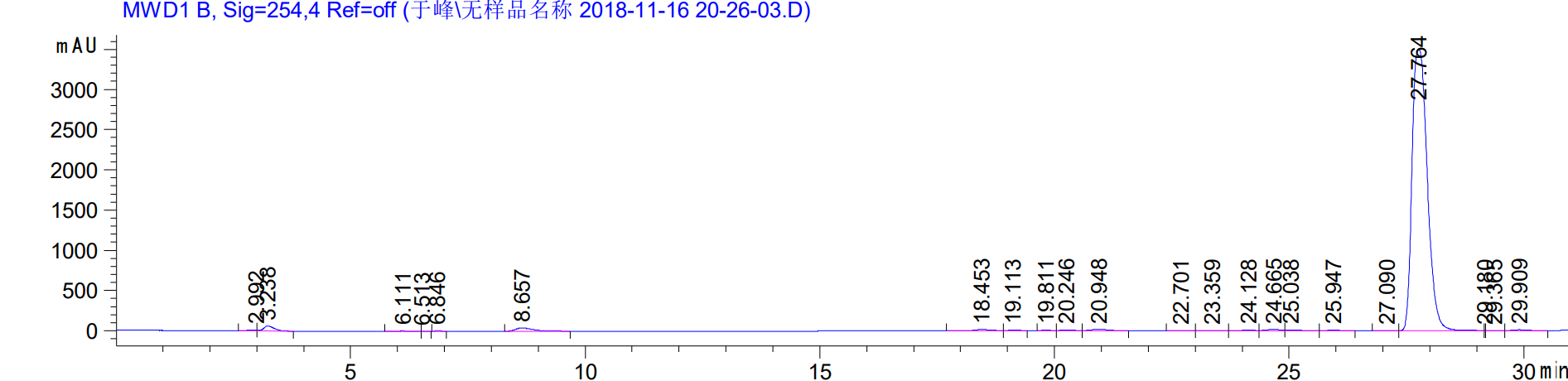


**Figure S19**. HPLC signal of methyl 1,4-dihydro-4-oxo-2-quinolinecarboxylate (**4**)

**Table S4.** Insecticidal activity of compound **4**

| **Conc. (ppm)** | **Mean mortality (%) with exposure time (h)** | | | | | |
| --- | --- | --- | --- | --- | --- | --- |
|  | **Contact toxicity assay** | | | **Feeding toxicity assay** | | |
|  | **24** | **48** | **72** | **24** | **48** | **72** |
| **50** | 25.00±1.93^b^ | 42.50±3.19^b^ | 87.50±7.87^a^ | 40.83±8.76^b^ | 85.00±6.93^ab^ | 100.00±0.00^a^ |
| **25** | 19.17±5.00^bc^ | 30.00±4.71^b^ | 56.67±15.63^b^ | 38.33±5.77^b^ | 71.67±7.93^c^ | 92.50±5.69^a^ |
| **12.5** | 15.83±3.19^c^ | 24.99±5.77^cd^ | 52.50±1287^b^ | 30.83±7.39^b^ | 44.17±8.76^d^ | 76.66±11.22^b^ |
| **6.25** | 11.67±1.92^c^ | 18.33±3.33^d^ | 48.33±8.81^b^ | 29.17±14.2^b^ | 40.00±13.61^d^ | 66.67±5.44^b^ |
| **PC** | 80.83±5.69^a^ | 97.50±3.19^a^ | 100.00±0.00^a^ | 88.33±4.30^a^ | 100.0±0.00^a^ | 100.0±0.00^a^ |
| **CK** | 3.33±2.72^d^ | 5.83±1.67^e^ | 13.33±0.00^c^ | 5.00±1.92^c^ | 8.33±1.92^e^ | 15.83±1.67^c^ |
| **Statics summary** | |  |  |  |  |  |
| **S.S** | 15507 | 20807 | 18949 | 15026 | 22646 | 20513 |
| **M.S** | 3101.5 | 4161.7 | 3789.8 | 3005 | 4529.2 | 4102.7 |
| **df** | 5 | 5 | 5 | 5 | 5 | 5 |
| **f** | 225.7*** | 227.93*** | 41.35*** | 46.2*** | 72.1*** | 129.1*** |

Data in the columns is described as mean values ± standard deviation with various superscripts is significantly

different according to DMRT ( P> 0.05). S.S (Sum of square); Df (Degree of freedom); M.S (Mean square); F

(Significance); PC (Postive Control); CK (Check); *** (level of significance).


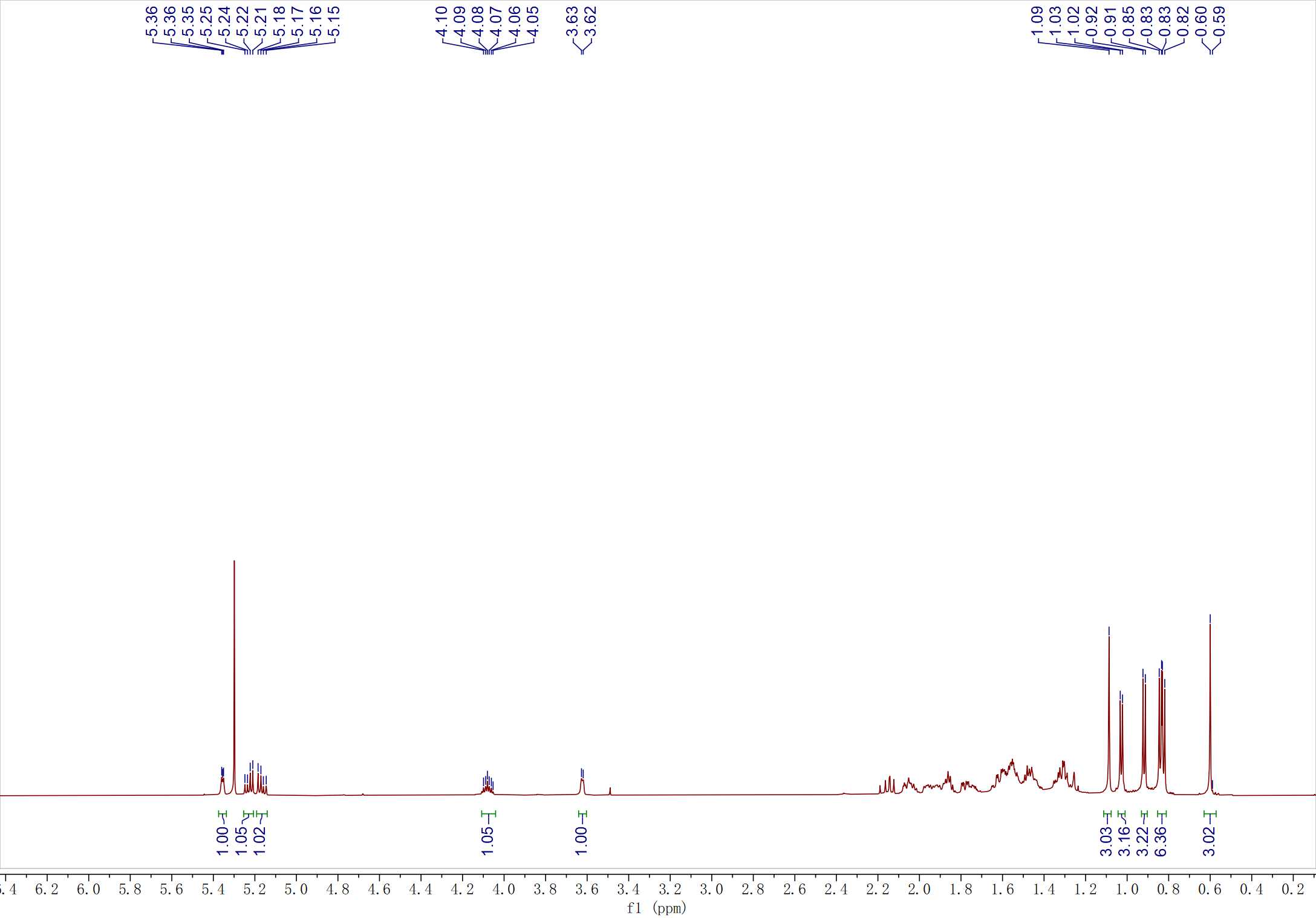


Figure S20. ^1^H NMR spectrum (CDCl3, 600 MHz) of cerevisterol (**5**)


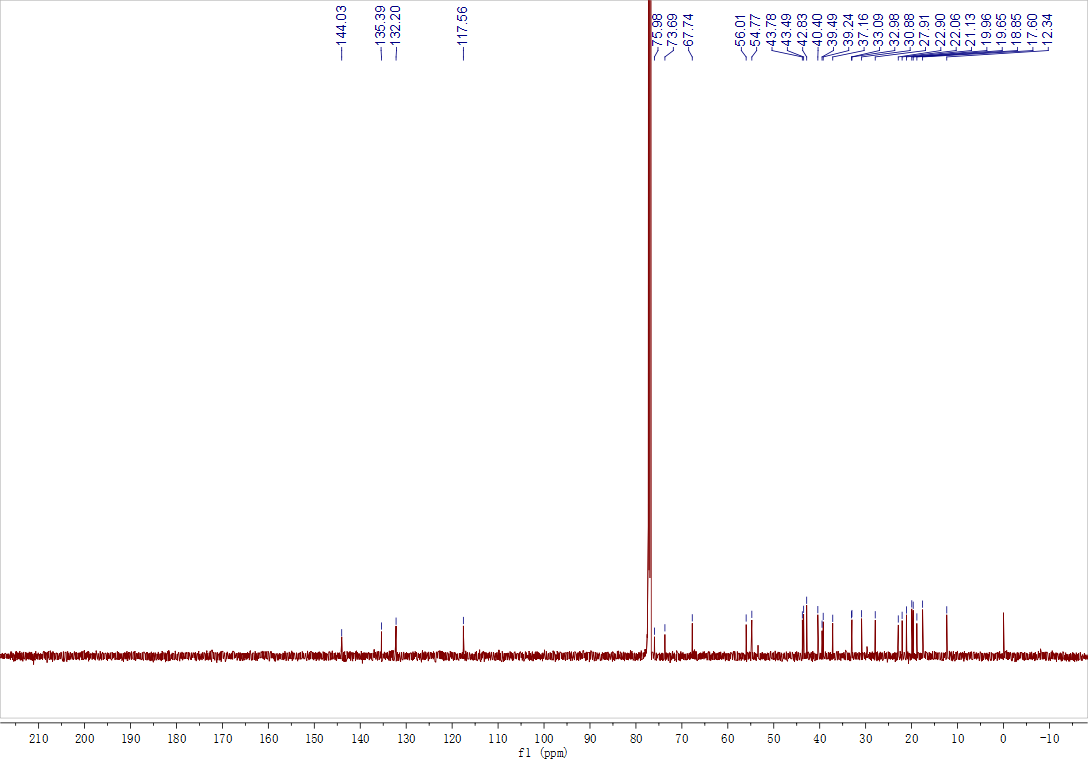


Figure S21. ^13^C NMR spectrum (CDCl3, 600 MHz) of cerevisterol (**5**)

**Figure S22**. ESI-MS data of cerevisterol (**5**)

**Table S5.** Insecticidal activity of compound **5**

| **Conc. (ppm)** | **Mean mortality (%) with exposure time (h)** | | | | | |
| --- | --- | --- | --- | --- | --- | --- |
|  | **Contact toxicity assay** | | | **Feeding toxicity assay** | | |
|  | **24** | **48** | **72** | **24** | **48** | **72** |
| **50** | 41.67±6.38^b^ | 44.17±7.39^b^ | 53.33±16.55^b^ | 30.83±18.53^b^ | 42.50±7.39^b^ | 70.83±11.01^b^ |
| **25** | 30.83±7.39^bc^ | 36.67±7.20^bc^ | 50.83±8.76^b^ | 25.00±5.77^bc^ | 40.00±7.20^b^ | 66.67±6.08^bc^ |
| **12.5** | 21.66±12.83^c^ | 25.00±15.52^bcd^ | 45.00±11.71^bc^ | 21.67±3.33^bc^ | 38.33±19.72^b^ | 56.67±11.85^bc^ |
| **6.25** | 19.17±12.83^cd^ | 19.16±10.31^cd^ | 25.17±8.33^cd^ | 19.17±13.71^bc^ | 30.33±23.73^bc^ | 49.17±8.76^c^ |
| **PC** | 80.83±5.69^a^ | 97.50±3.19^a^ | 100.00±0.00^a^ | 88.33±4.30^a^ | 100.0±0.00^a^ | 100.0±0.00^a^ |
| **CK** | 3.33±2.72^d^ | 5.83±1.67^d^ | 13.33±0.00^d^ | 5.00±2.72^c^ | 8.33±1.92^c^ | 15.83±1.67^d^ |
| **Statics summary** | |  |  |  |  |  |
| **S.S** | 14270 | 20553 | 17770 | 16893 | 18287 | 15362 |
| **M.S** | 2854.1 | 4110.7 | 3554.19 | 3378.8 | 3657.4 | 3072.5 |
| **df** | 5 | 5 | 5 | 5 | 5 | 5 |
| **f** | 44.35*** | 52.25*** | 38.26*** | 33.9*** | 21.1*** | 46.68*** |

Data in the columns is described as mean values ± standard deviation with various superscripts is significantly

different according to DMRT ( P> 0.05). S.S (Sum of square); Df (Degree of freedom); M.S (Mean square); F

(Significance); PC (Postive Control); CK (Check); *** (level of significance).


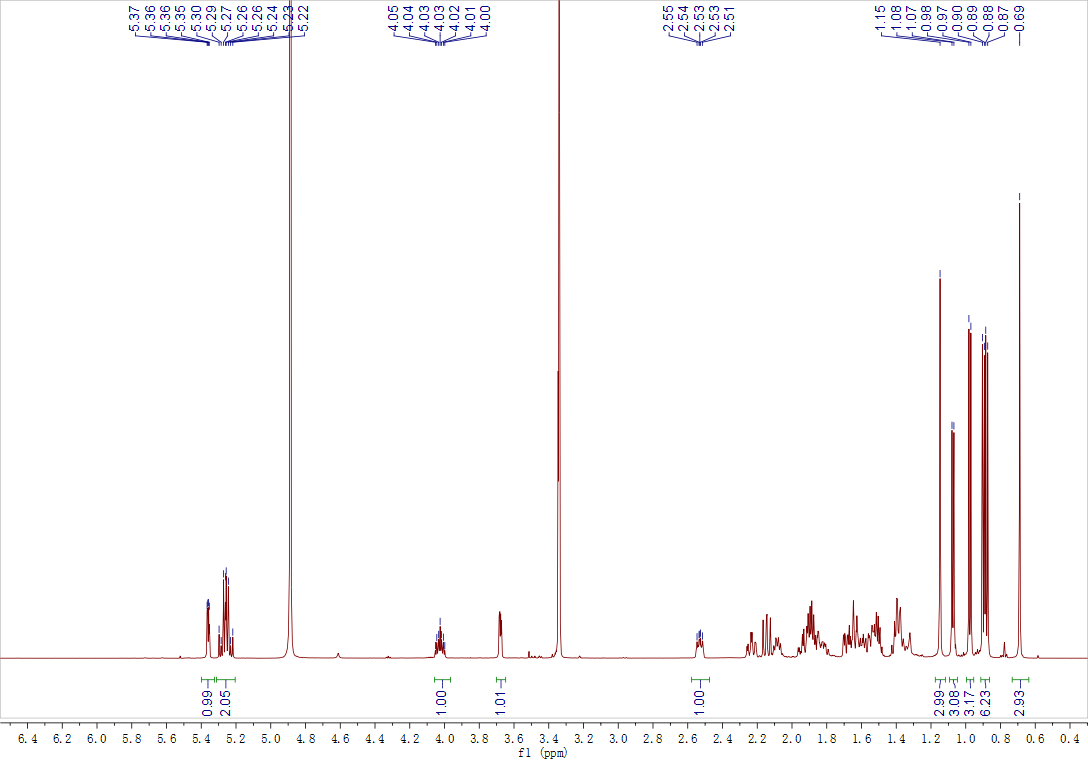


Figure S23. ^1^H NMR spectrum (MeOD, 600 MHz) of 9-hydroxycerevisterol (**6**)


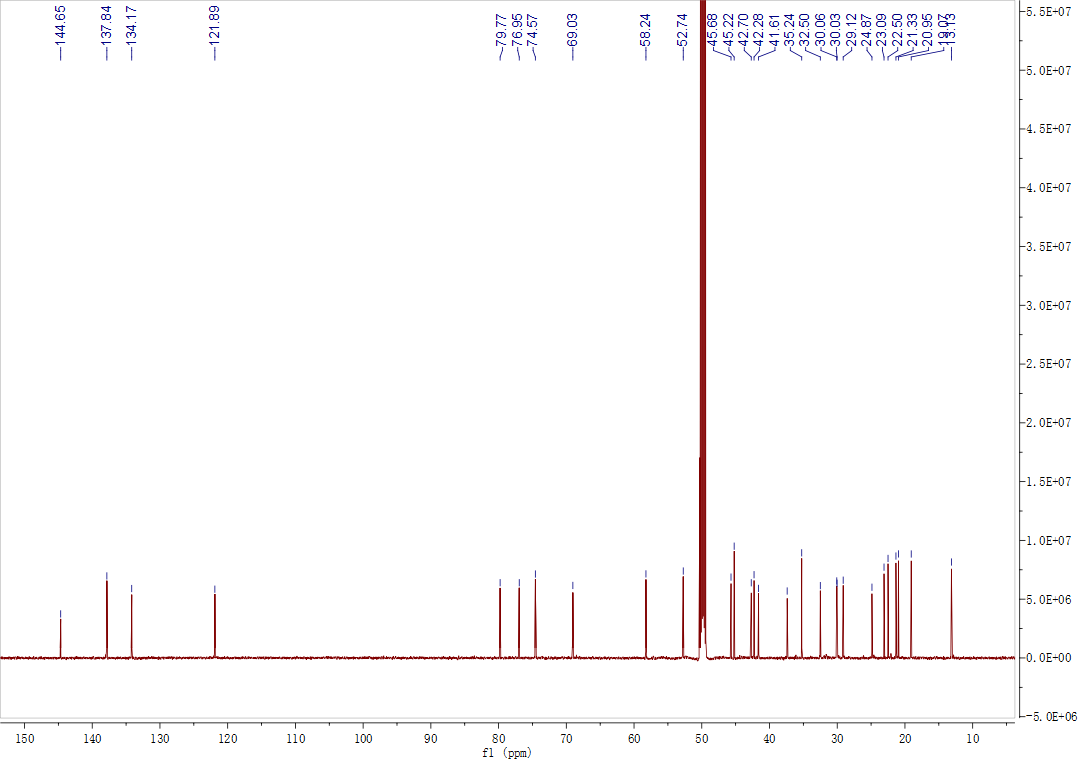


Figure S24. ^13^C NMR spectrum (MeOD, 600 MHz) of 9-hydroxycerevisterol (**6**)

**Figure S25**. HR-ESI-MS data of 9-hydroxycerevisterol (**6**)


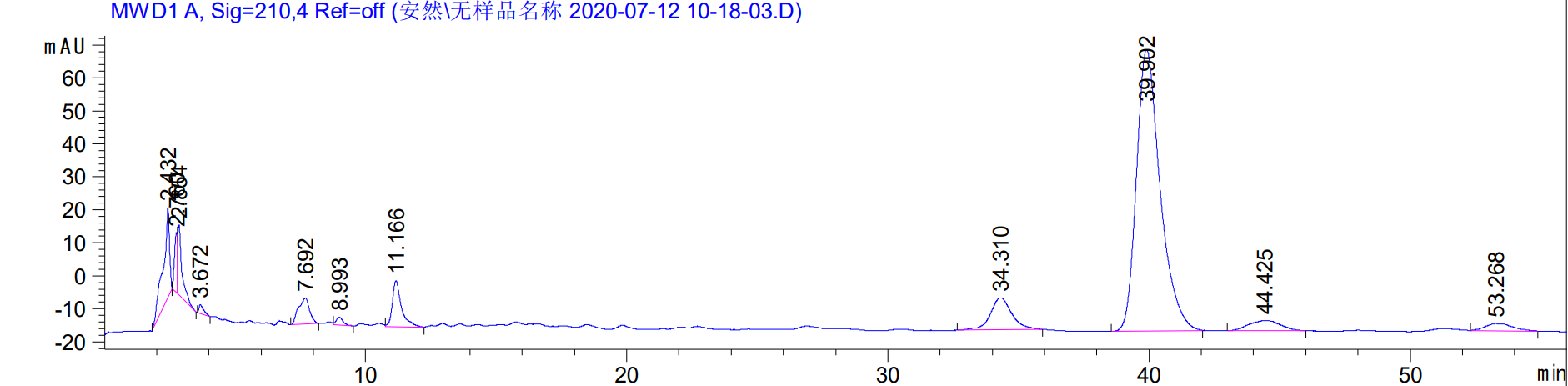


**Figure S26**. HPLC signal of 9-hydroxycerevisterol (**6**)

**Table S6.** Insecticidal activity of compound **6**

| **Conc. (ppm)** | **Mean mortality (%) with exposure time (h)** | | | | | |
| --- | --- | --- | --- | --- | --- | --- |
|  | **Contact toxicity assay** | | | **Feeding toxicity assay** | | |
|  | **24** | **48** | **72** | **24** | **48** | **72** |
| **50** | 19.17±8.33^b^ | 25.00±8.81^b^ | 36.67±4.71^b^ | 19.17±9.18^b^ | 25.00±4.30^b^ | 41.67±10.36^b^ |
| **25** | 14.17±5.69^bc^ | 15.83±6.87^bc^ | 28.34±5.77^bc^ | 17.50±11.01^b^ | 23.33±6.08^b^ | 38.33±4.30^b^ |
| **12.5** | 8.33±4.30^bc^ | 15.00±3.30^bc^ | 22.50±7.39^cd^ | 9.17±4.19^b^ | 18.33±7.93^bc^ | 28.33±4.30^bc^ |
| **6.25** | 6.67±2.72^c^ | 11.67±1.92^c^ | 19.17±3.19^cd^ | 5.00±4.30^b^ | 9.99±8.16^c^ | 21.67±8.82^c^ |
| **PC** | 80.83±5.69^a^ | 97.50±3.19^a^ | 100.00±0.00^a^ | 88.33±4.30^a^ | 100.0±0.00^a^ | 100.0±0.00^a^ |
| **CK** | 3.33±2.72^c^ | 5.83±1.67^c^ | 13.33±0.00^d^ | 5.00±2.72^b^ | 8.33±1.92^c^ | 15.83±1.67^c^ |
| **Statics summary** | |  |  |  |  |  |
| **S.S** | 17203 | 23651 | 20528 | 20585 | 23833 | 18264 |
| **M.S** | 3440.7 | 4730.4 | 4105.6 | 4117.1 | 4776.7 | 3724.9 |
| **df** | 5 | 5 | 5 | 5 | 5 | 5 |
| **f** | 123.17*** | 177.19*** | 204.59*** | 93.6*** | 151.7*** | 99.35*** |

Data in the columns is described as mean values ± standard deviation with various superscripts is significantly

different according to DMRT ( P> 0.05). S.S (Sum of square); Df (Degree of freedom); M.S (Mean square); F

(Significance); PC (Postive Control); CK (Check); *** (level of significance).


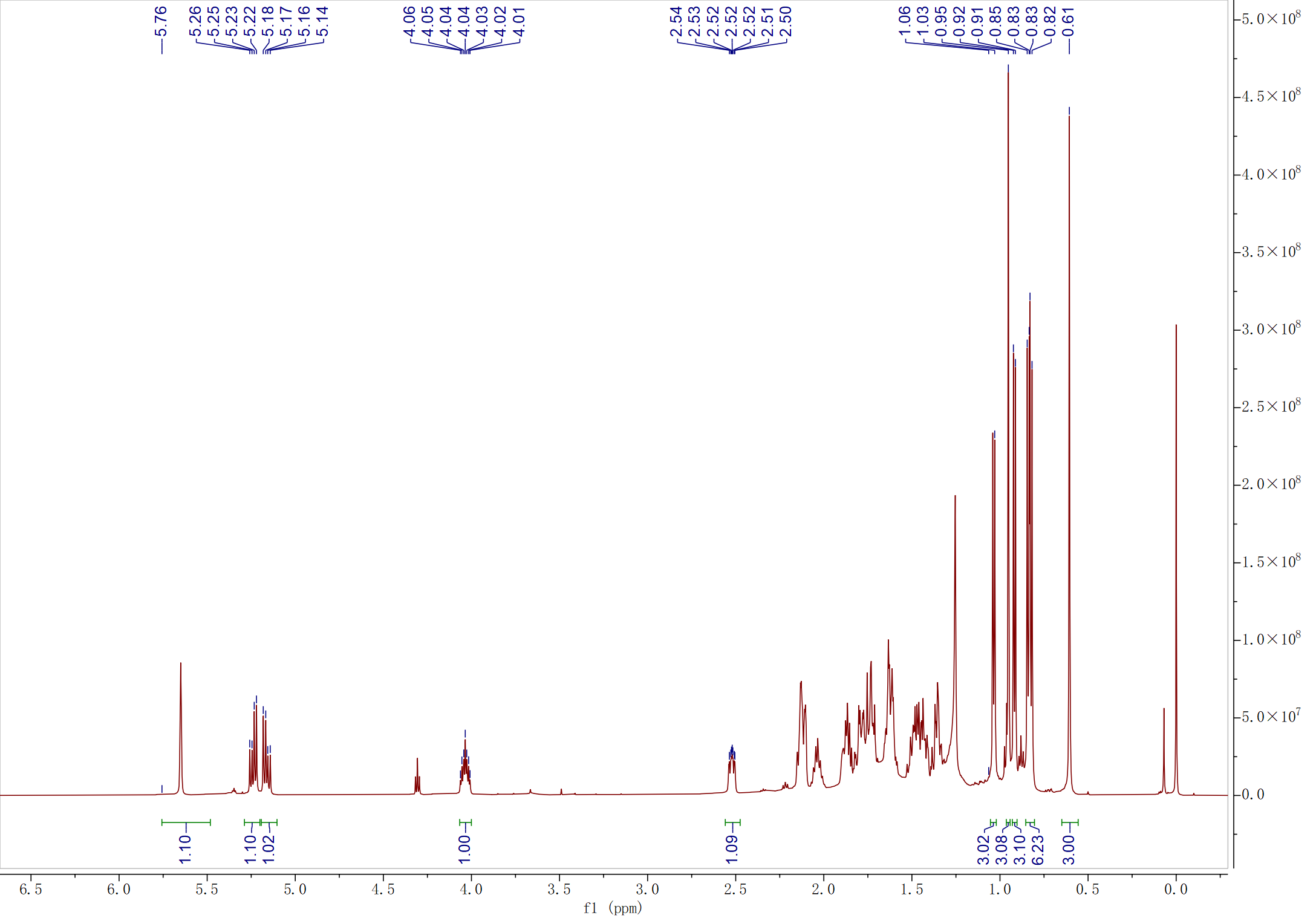


Figure S27. ^1^H NMR spectrum (CDCl3, 600 MHz) of 6-dehydrocerevisterol (**7**)


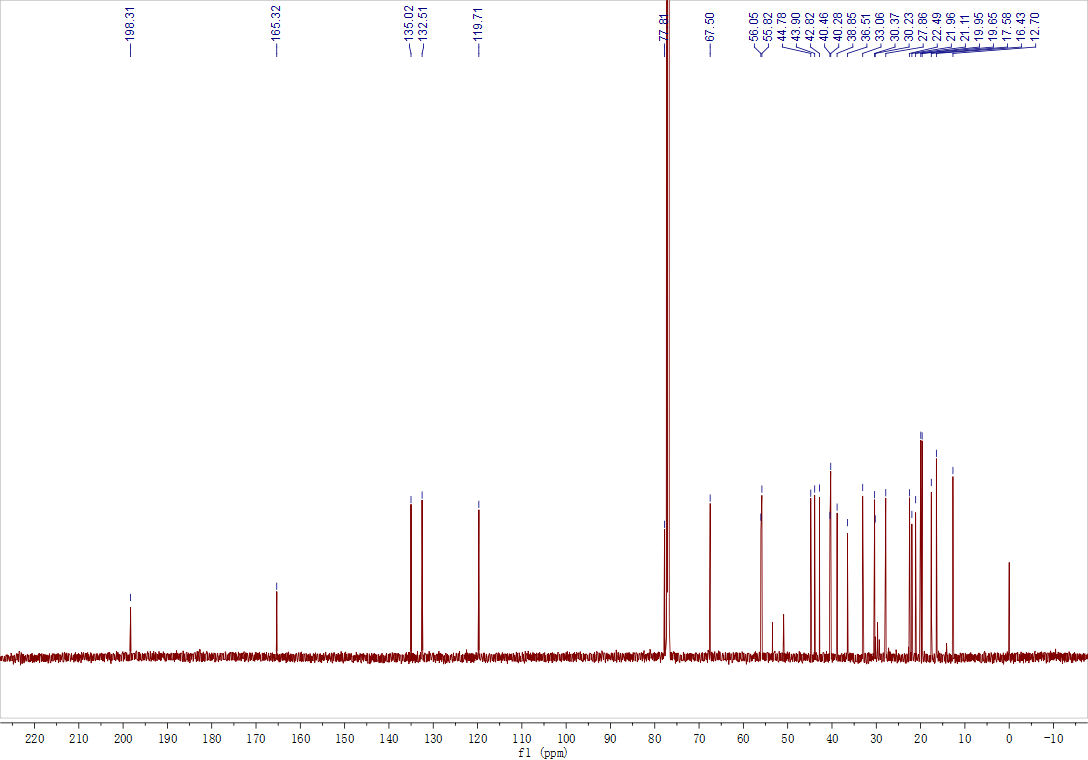


Figure S28. ^13^C NMR spectrum (CDCl3, 600 MHz) of 6-dehydrocerevisterol (**7**)

**Figure S29**. ESI-MS data of 6-dehydrocerevisterol (**7**)


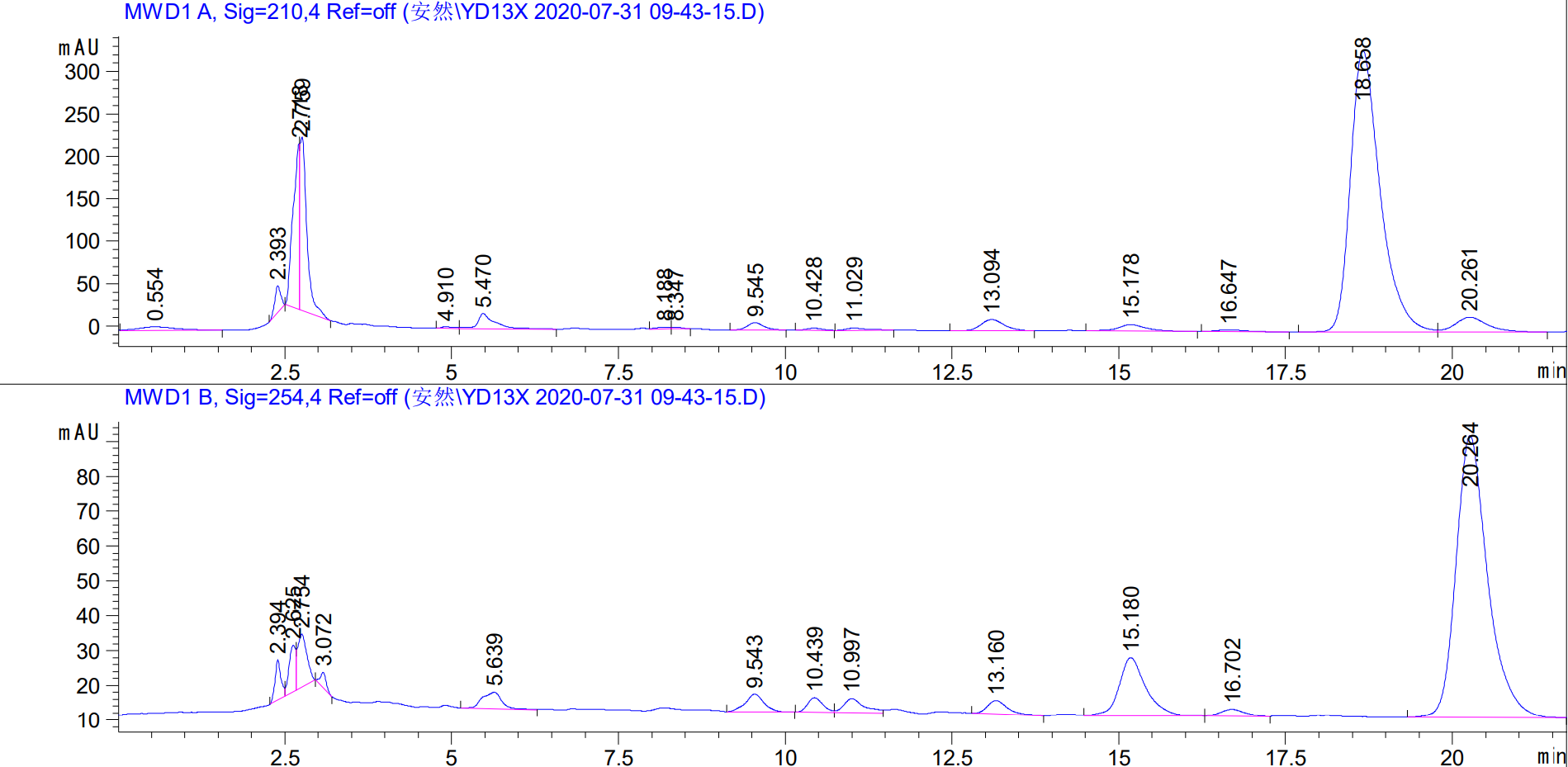


**Figure S30**. HPLC signal of 6-dehydrocerevisterol (**7**)

**Table S7**: Insecticidal activity of compound **7**

| **Conc. (ppm)** | **Mean mortality (%) with exposure time (h)** | | | | | |
| --- | --- | --- | --- | --- | --- | --- |
|  | **Contact toxicity assay** | | | **Feeding toxicity assay** | | |
|  | **24** | **48** | **72** | **24** | **48** | **72** |
| **50** | 26.67±5.44^b^ | 45.00±8.81^b^ | 71.67±4.71^b^ | 40.83±8.76^b^ | 85.00±6.94^ab^ | 100.00±0.00^a^ |
| **25** | 19.99±10.53^b^ | 35.00±12.32^b^ | 65.83±5.77^bc^ | 38.33±5.77^b^ | 71.67±7.93^c^ | 92.50±5.69^a^ |
| **12.5** | 18.33±5.77^b^ | 35.00±11.05^b^ | 47.50±7.39^bc^ | 30.83±7.39^b^ | 44.17±8.77^d^ | 76.67±11.22^b^ |
| **6.25** | 15.00±4.30^b^ | 27.49±6.87^b^ | 44.17±3.19^c^ | 29.17±14.2^b^ | 40.00±13.61^d^ | 66.67±5.44^b^ |
| **PC** | 80.83±5.69^a^ | 97.50±3.19^a^ | 100.00±0.00^a^ | 88.33±4.30^a^ | 100.0±0.00^a^ | 100.0±0.00^a^ |
| **CK** | 3.33±2.72^c^ | 5.83±1.67^c^ | 13.33±0.00^d^ | 5.00±2.72^c^ | 8.33±1.92^e^ | 15.83±1.67^c^ |
| **Statics summary** | |  |  |  |  |  |
| **S.S** | 14902 | 18796 | 17216 | 15026 | 22646 | 20513 |
| **M.S** | 2980.46 | 3759.3 | 3443.3 | 3005.2 | 4529.2 | 4102.7 |
| **df** | 5 | 5 | 5 | 5 | 5 | 5 |
| **f** | 76.95*** | 54.75*** | 25.67*** | 46.2*** | 72.1*** | 129.1*** |

Data in the columns is described as mean values ± standard deviation with various superscripts is significantly

different according to DMRT ( P> 0.05). S.S (Sum of square); Df (Degree of freedom); M.S (Mean square); F

(Significance); PC (Postive Control); CK (Check); *** (level of significance).


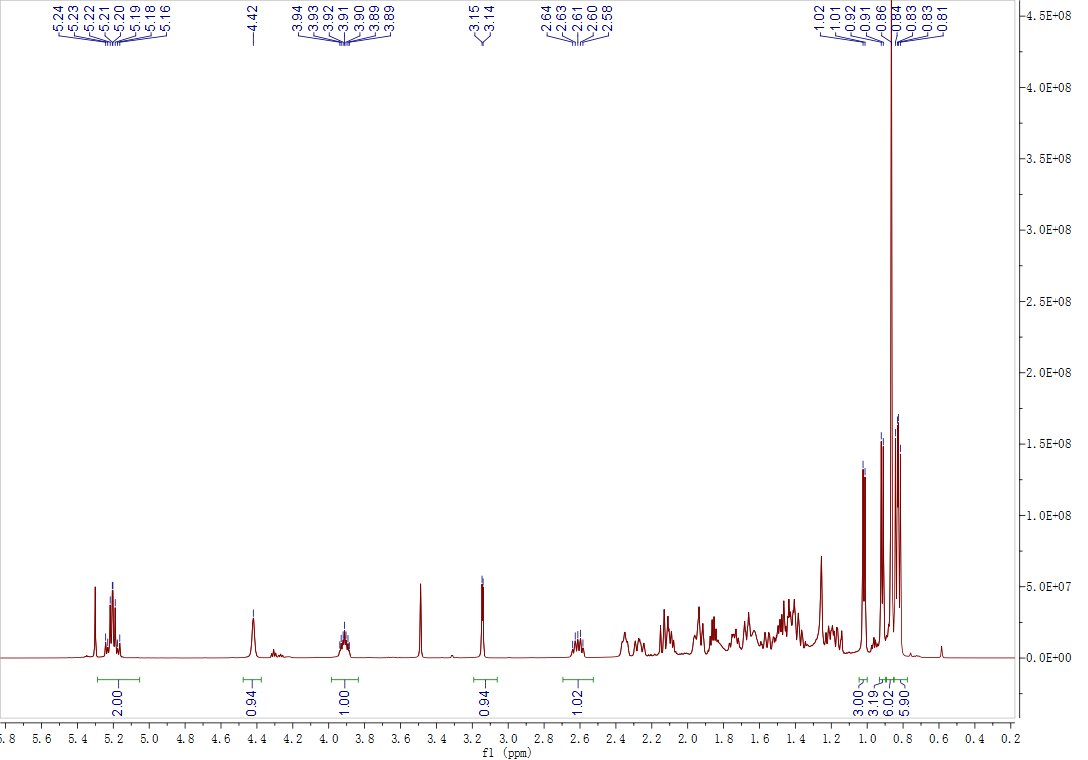


Figure S31. ^1^H NMR spectrum (CDCl3, 600 MHz) of (22E,24R)-ergosta-8(14),22-diene-3β,5α,6β,7α-tetrol (**8**)


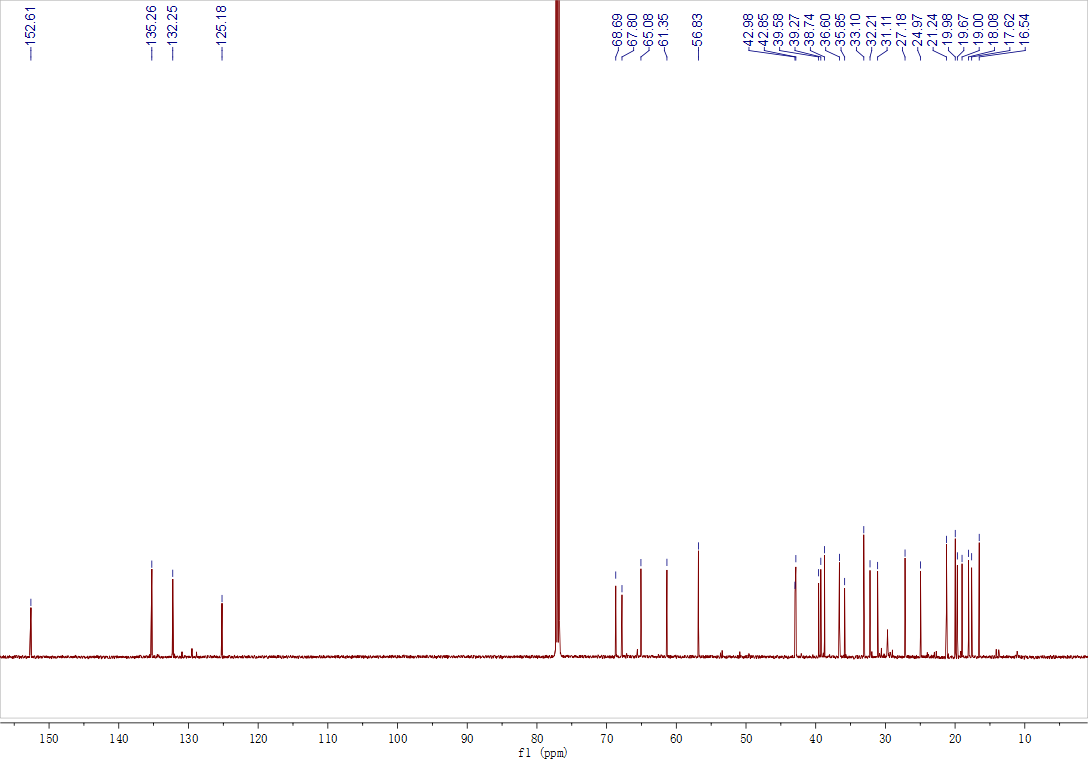


Figure S32. ^13^C NMR spectrum (CDCl3, 600 MHz) of (22E,24R)-ergosta-8(14),22-diene-3β,5α,6β,7α-tetrol (**8**)

**Figure S33**. ESI-MS data of (22E,24R)-ergosta-8(14),22-diene-3β,5α,6β,7α-tetrol (**8**)


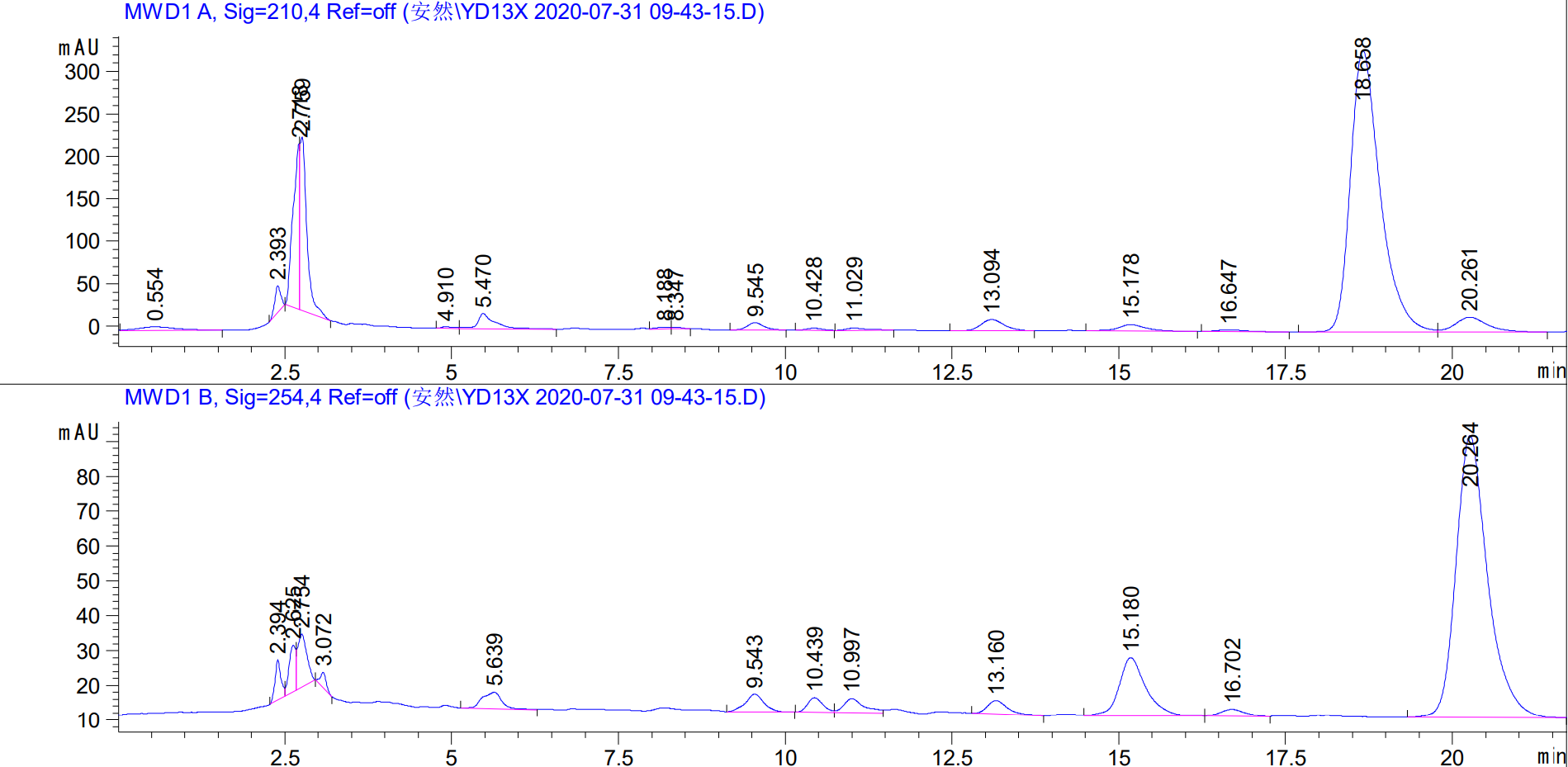


**Figure S34**. HPLC signal of (22E,24R)-ergosta-8(14),22-diene-3β,5α,6β,7α-tetrol (**8**)

**Table S8**. Insecticidal activity of compound **8**

| **Conc. (ppm)** | **Mean mortality (%) with exposure time (h)** | | | | | |
| --- | --- | --- | --- | --- | --- | --- |
|  | **Contact toxicity assay** | | | **Feeding toxicity assay** | | |
|  | **24** | **48** | **72** | **24** | **48** | **72** |
| **50** | 43.33±13.33^b^ | 56.67±9.82^b^ | 61.67±10.37^b^ | 27.50±8.33^b^ | 45.83±8.76^b^ | 90.00±11.54^ab^ |
| **25** | 41.67±14.53^bc^ | 49.17±11.35^bc^ | 57.50±15.24^b^ | 17.50±5.69^bc^ | 30.83±7.39^bc^ | 81.67±4.30^b^ |
| **12.5** | 30.83±9.18^bc^ | 42.50±5.69^bc^ | 56.67±13.05^b^ | 16.67±9.02^bc^ | 25.83±6.30^c^ | 57.50±9.17^c^ |
| **6.25** | 19.16±10.32^cd^ | 28.33±15.99^c^ | 49.17±3.7^b^ | 15.00±12.32^bc^ | 22.50±11.01^cd^ | 45.00±9.99^C^ |
| **PC** | 80.83±5.69^a^ | 97.50±3.19^a^ | 100.00±0.00^a^ | 88.33±4.30^a^ | 100.0±0.00^a^ | 100.0±0.0^a^ |
| **CK** | 3.33±2.72^d^ | 5.83±1.67^d^ | 13.33±0.00^c^ | 5.00±2.72^c^ | 8.33±1.92^d^ | 15.83±1.67^d^ |
| **Statics summary** | |  |  |  |  |  |
| **S.S** | 13885 | 18843 | 15349 | 18305 | 20880 | 20004 |
| **M.S** | 2777.15 | 3768.74 | 3069.85 | 3661.1 | 4176.2 | 4000.9 |
| **df** | 5 | 5 | 5 | 5 | 5 | 5 |
| **f** | 26.90*** | 42.98*** | 26.38*** | 61.5*** | 84.6*** | 70.9*** |

Data in the columns is described as mean values ± standard deviation with various superscripts is significantly

different according to DMRT ( P> 0.05). S.S (Sum of square); Df (Degree of freedom); M.S (Mean square); F

(Significance); PC (Postive Control); CK (Check); *** (level of significance).


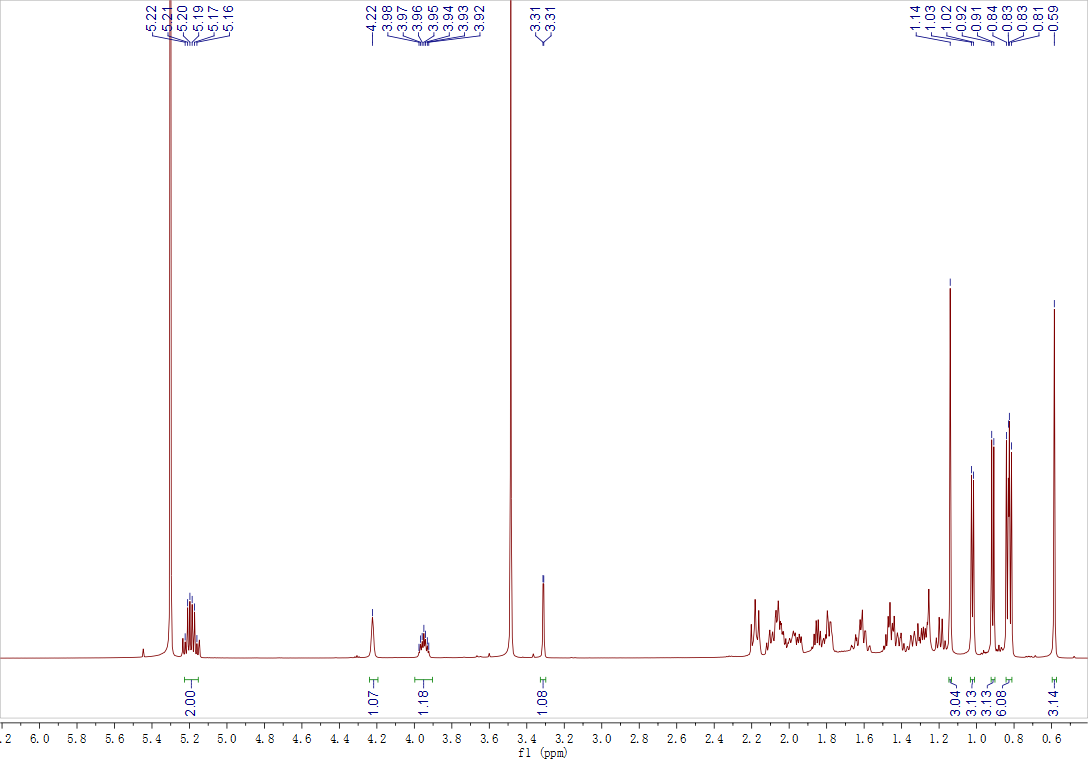


Figure S35. ^1^H NMR spectrum (CDCl3, 600 MHz) of melithasterol B (**9**)


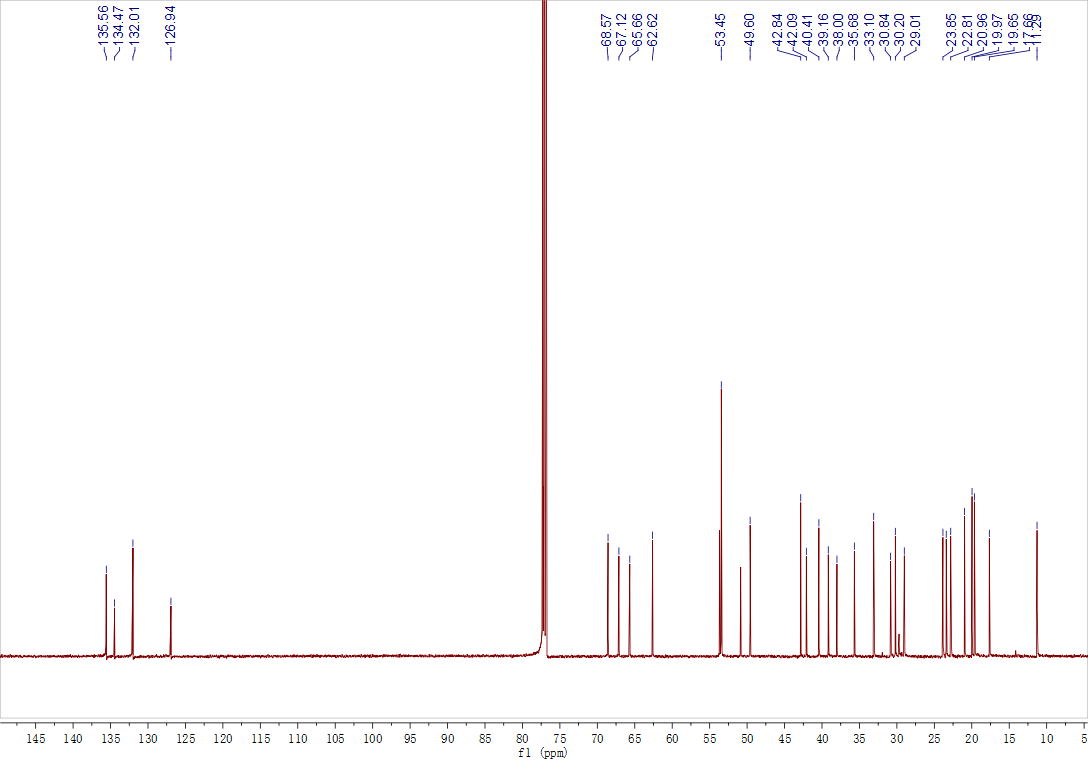


Figure S36. ^13^C NMR spectrum (CDCl3, 600 MHz) of melithasterol B (**9**)

**Figure S37**. ESI-MS data of melithasterol B (**9**)

**Table S9**. Insecticidal activity of compound **9**

| **Conc. (ppm)** | **Mean mortality (%) with exposure time (h)** | | | | | |
| --- | --- | --- | --- | --- | --- | --- |
|  | **Contact toxicity assay** | | | **Feeding toxicity assay** | | |
|  | **24** | **48** | **72** | **24** | **48** | **72** |
| **50** | 33.33±13.87^b^ | 51.67±16.87^b^ | 66.67±18.39^b^ | 22.50±4.19^b^ | 50.83±11.98^b^ | 64.17±23.62^b^ |
| **25** | 30.00±6.08^b^ | 35.83±8.34^bc^ | 55.00±13.61^bc^ | 11.67±4.30^c^ | 25.83±12.87^c^ | 38.33±9.62^bc^ |
| **12.5** | 21.67±4.30^bc^ | 24.17±5.69^cd^ | 48.33±6.93^c^ | 11.67±5.77^c^ | 21.67±4.73^cd^ | 36.67±11.22^c^ |
| **6.25** | 7.49±5.00^cd^ | 18.33±11.87^cd^ | 40.83±4.30^c^ | 10.00±7.20^c^ | 20.00±4.30^cd^ | 36.67±8.17^c^ |
| **PC** | 80.83±5.69^a^ | 97.50±3.19^a^ | 100.00±0.00^a^ | 88.33±4.30^a^ | 100.0±0.00^a^ | 100.0±0.00^a^ |
| **CK** | 3.33±2.72^d^ | 5.84±1.67^d^ | 13.33±0.00^d^ | 5.00±1.92^c^ | 8.33±1.92^d^ | 15.83±1.67^c^ |
| **Statics summary** | |  |  |  |  |  |
| **S.S** | 15520 | 21358 | 16548 | 19990 | 22508 | 17391 |
| **M.S** | 3104.1 | 4271.8 | 3309.6 | 3898.1 | 4501.6 | 3478.4 |
| **df** | 5 | 5 | 5 | 5 | 5 | 5 |
| **f** | 59.51*** | 48.39*** | 64.79*** | 261.6*** | 76.4*** | 24.7*** |

Data in the columns is described as mean values ± standard deviation with various superscripts is significantly

different according to DMRT ( P> 0.05). S.S (Sum of square); Df (Degree of freedom); M.S (Mean square); F

(Significance); PC (Postive Control); CK (Check); *** (level of significance).


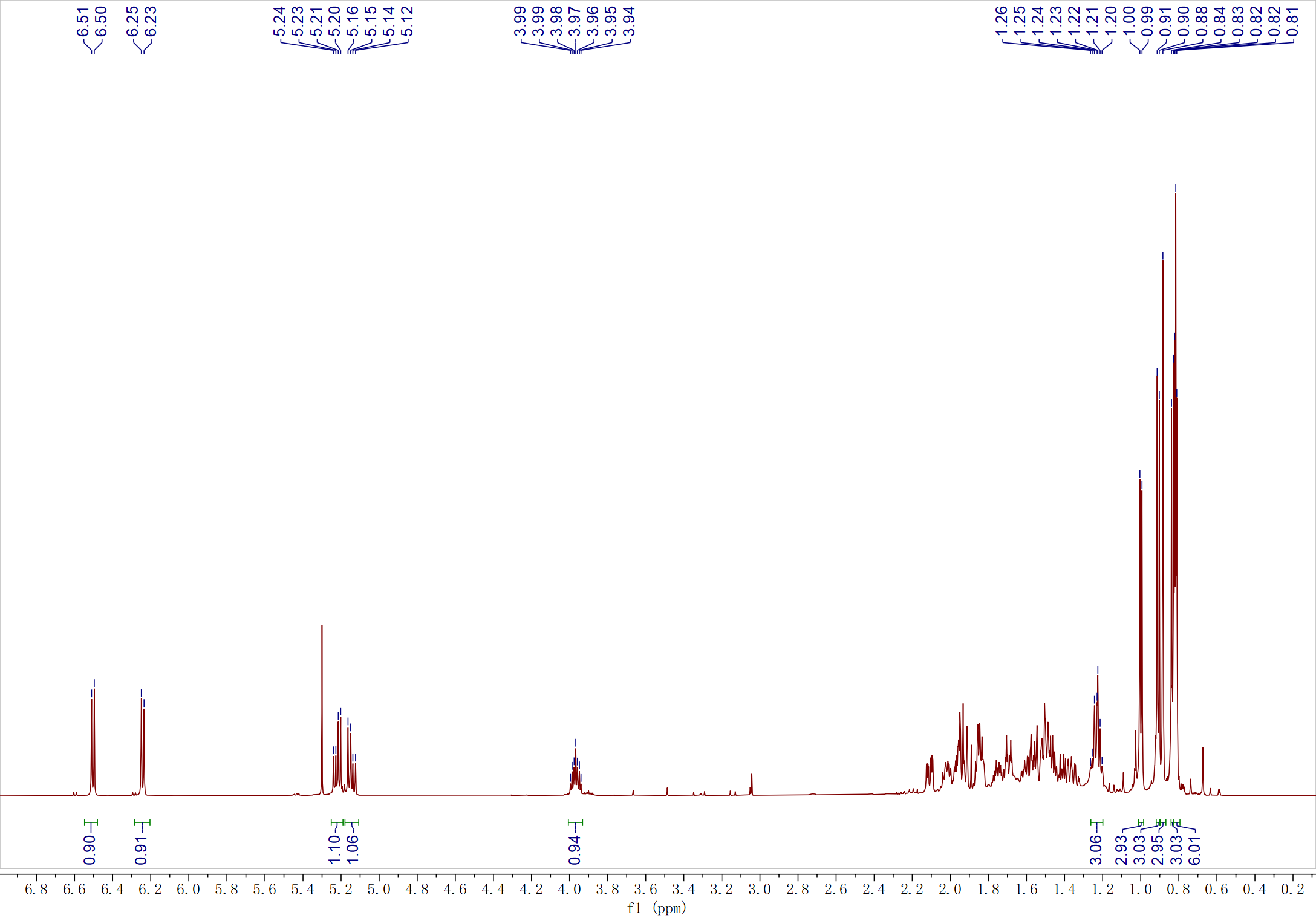


Figure S38. ^1^H NMR spectrum (CDCl_3_, 600 MHz) of ergosterol peroxide (**10**)


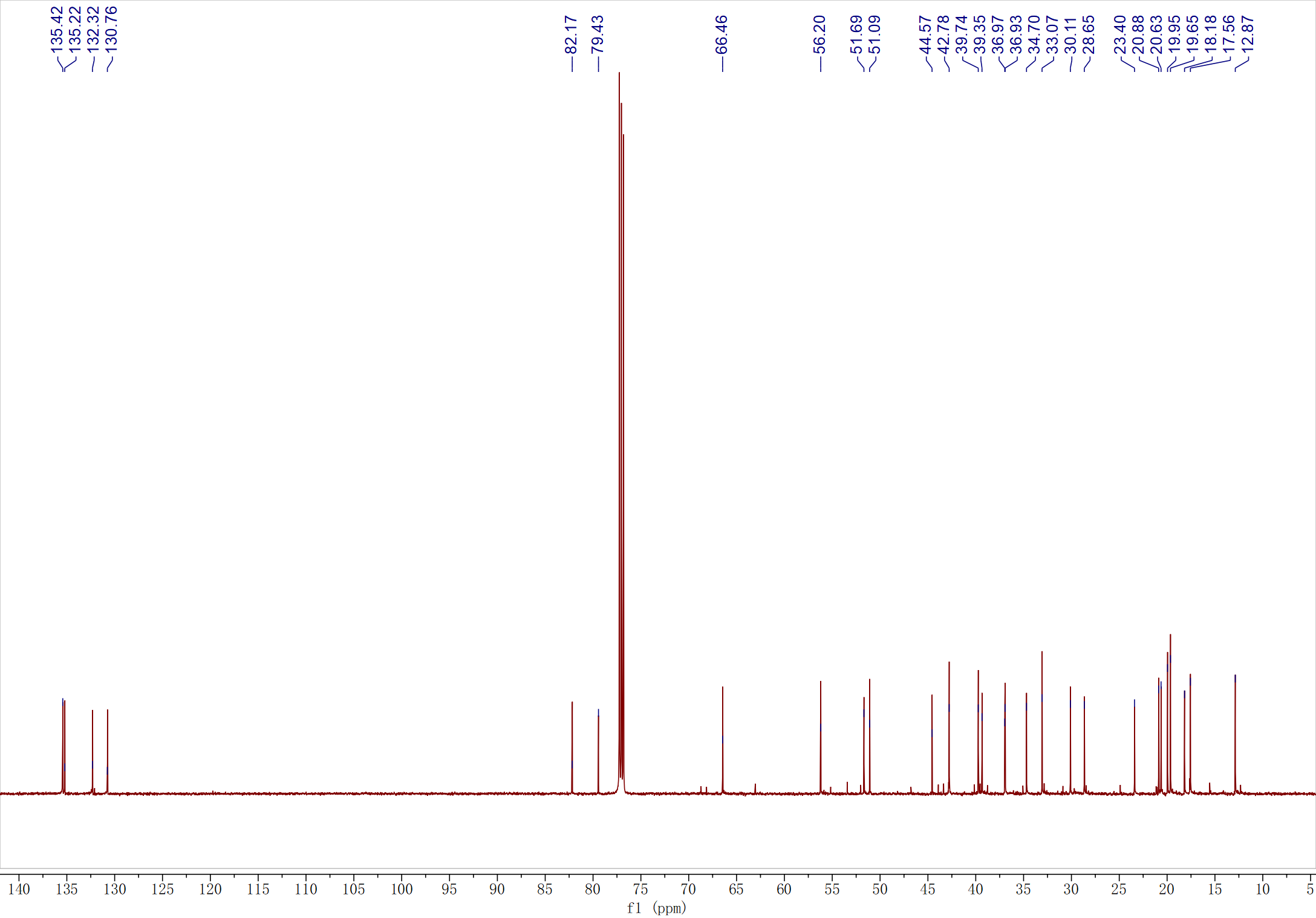


Figure S39. ^13^C NMR spectrum (CDCl_3_, 600 MHz) of ergosterol peroxide (**10**)

**Figure S40**. ESI-MS data of ergosterol peroxide (**10**)

**Table S10**: Insecticidal activity of compound **10**

| **Conc. (ppm)** | **Mean mortality (%) with exposure time (h)** | | | | | |
| --- | --- | --- | --- | --- | --- | --- |
|  | **Contact toxicity assay** | | | **Feeding toxicity assay** | | |
|  | **24** | **48** | **72** | **24** | **48** | **72** |
| **50** | 15.00±8.92^b^ | 44.17±6.87^b^ | 56.67±15.15^b^ | 15.83±4.19^b^ | 45.00±10.36^b^ | 80.83±12.8^ab^ |
| **25** | 9.17±10.32^b^ | 34.17±7.39^bc^ | 45.83±15.48^b^ | 15.00±4.30^b^ | 30.00±8.61^bc^ | 68.33±11.05^b^ |
| **12.5** | 4.17±4.19^b^ | 23.33±3.85^c^ | 42.50±23.93^bc^ | 11.67±5.77^b^ | 23.33±3.85^cd^ | 44.17±9.57^c^ |
| **6.25** | 3.33±2.72^b^ | 9.99±4.71^d^ | 40.84±8.76^bc^ | 10.00±7.20^b^ | 23.33±13.6^cd^ | 40.83±14.2^c^ |
| **PC** | 80.83±5.69^a^ | 97.50±3.19^a^ | 100.00±0.00^a^ | 88.33±4.30^a^ | 100.0±0.00^a^ | 100.0±0.00^a^ |
| CK | 3.33±2.72^b^ | 5.84±1.67^d^ | 13.33±0.00^c^ | 3.33±2.72^b^ | 5.83±1.67^e^ | 15.33±0.00^d^ |
| **Statics summary** | |  |  |  |  |  |
| **S.S** | 18481 | 22394 | 16186 | 19979 | 21066 | 18621 |
| **M.S** | 3696.3 | 4478.8 | 3237.3 | 3995 | 4213.2 | 3724.2 |
| **df** | 5 | 5 | 5 | 5 | 5 | 5 |
| **f** | 88.04*** | 177.01*** | 17.35*** | 167.0*** | 65.6*** | 38.19*** |

Data in the columns is described as mean values ± standard deviation with various superscripts is significantly

different according to DMRT ( P> 0.05). S.S (Sum of square); Df (Degree of freedom); M.S (Mean square); F

(Significance); PC (Postive Control); CK (Check); *** (level of significance).


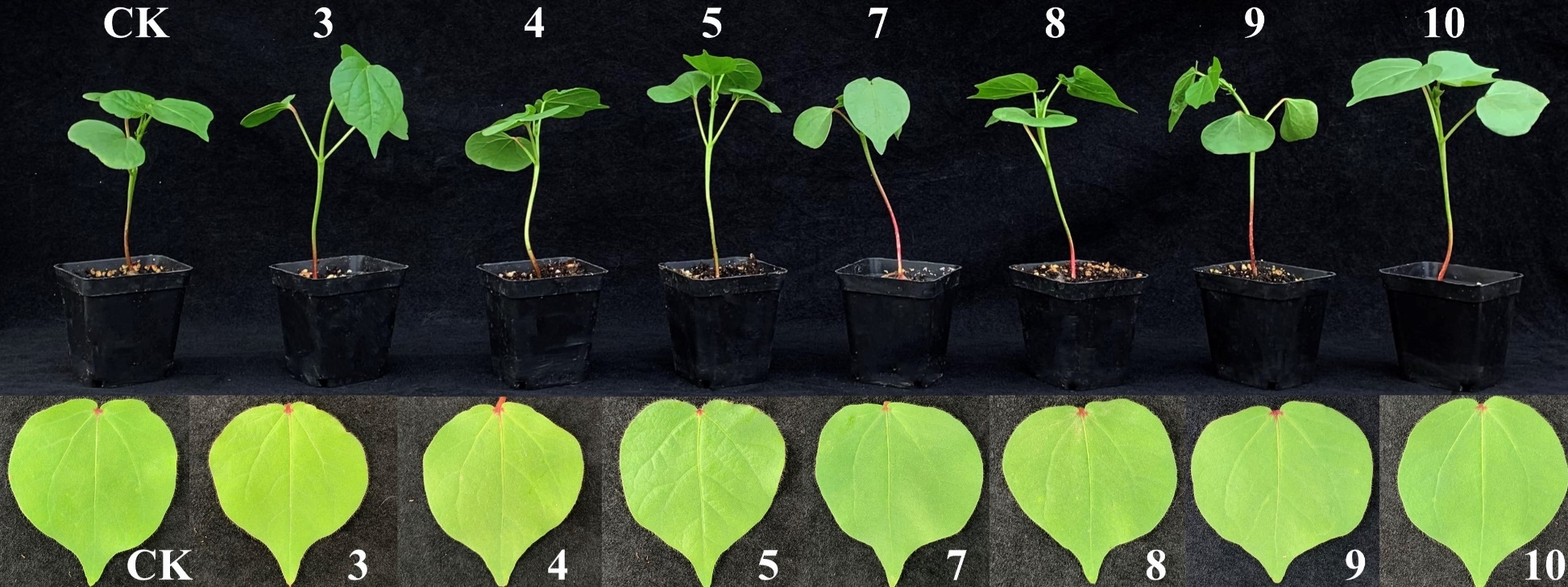


**Figure S41**. Effects of active compounds on cotton at 100 µg/mL

**Table S11.** Effects of active compounds on cotton height at 100 µg/mL

| **Day** | **Compound 3** | **Compound 4** | **Compound 5** | **Compound 7** | **Compound 8** | **Compound 9** | **Compound 10** | **CK** |
| --- | --- | --- | --- | --- | --- | --- | --- | --- |
| **0** | 12.97±0.3^b^ | 12.53±0.3^b^ | 12.36±0.3^b^ | 12.20±0.2^c^ | 12.63±0.3^c^ | 12.87±0.7^a^ | 12.13±0.3^c^ | 12.6±0.3^b^ |
| **7** | 13.6±0.4^ab^ | 13.8±0.6^ab^ | 13.40±0.4^ab^ | 13.80±0.2^b^ | 13.4±0.3^ab^ | 13.63±0.6^a^ | 13.76±0.4^b^ | 13.4±0.4^b^ |
| **14** | 14.60±0.3^a^ | 15.10±0.3^a^ | 14.43±0.2^a^ | 15.36±0.6^a^ | 14.37±0.3^a^ | 14.40±0.4^a^ | 15.13±0.4^a^ | 14.9±0.3^a^ |
|  | **Statistical Summary** | |  |  |  |  |  |  |
| **S.S** | 4.069 | 9.882 | 6.407 | 15.042 | 4.542 | 3.527 | 13.536 | 7.829 |
| **M.S** | 2.03 | 4.94 | 3.20 | 7.52 | 2.27 | 1.76 | 6.77 | 3.91 |
| **F** | 6.67* | 9.36* | 9.74* | 14.91** | 7.92* | 1.63 NS | 14.71** | 10.33* |
| **Df** | 2 | 2 | 2 | 2 | 2 | 2 | 2 | 2 |

Data in the columns presented as mean values ± standard error with various superscripts are significantly different according to DMRT（P > 0.05 ). S.S (Sum of square); M.S (Mean square); Df (Degree of freedom); F (Significance); CK (Check); *, ** (level of significance).


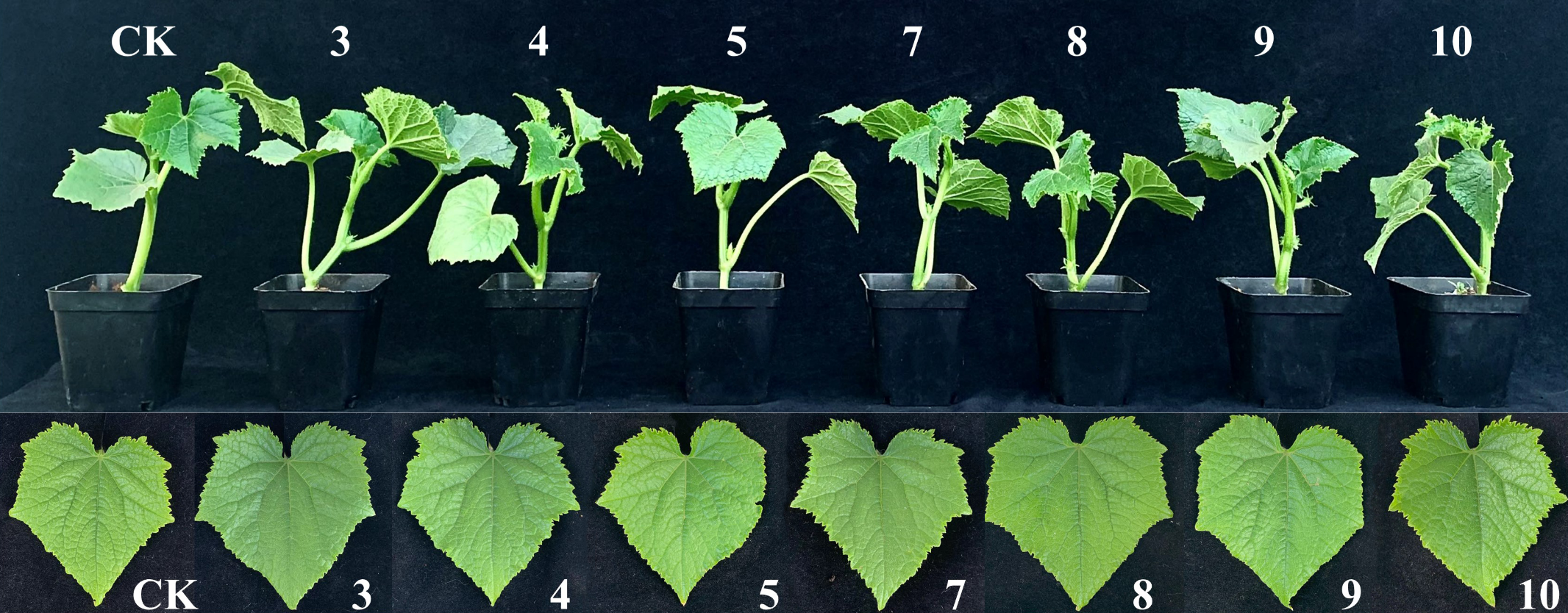


**Figure S42.** Effects of active compounds on cucumber at 100 µg/mL

**Table S12.** Effects of active compounds on cucumber height at 100 µg/mL

| **Day** | **Compound 3** | **Compound 4** | **Compound 5** | **Compound 7** | **Compound 8** | **Compound 9** | **Compound 10** | **CK** |
| --- | --- | --- | --- | --- | --- | --- | --- | --- |
| **0** | 7.23±0.3^c^ | 7.67±0.9^c^ | 7.40±0.3^c^ | 8.10±0.7^c^ | 7.80±0.4^c^ | 7.47±0.5^b^ | 8.40±0.6^c^ | 7.27±0.6^c^ |
| **7** | 11.57±0.6^b^ | 12.27±0.4^b^ | 12.07±0.3^b^ | 11.87±1.1^b^ | 12.00±0.6^b^ | 13.00±1.0^a^ | 12.33±0.7^b^ | 11.33±0.3^b^ |
| **14** | 15.17±0.5^a^ | 14.77±0.7^a^ | 15.13±0.6^a^ | 14.67±0.3^a^ | 15.23±0.8^a^ | 14.47±0.4^a^ | 14.60±0.3^a^ | 15.00±0.6^a^ |
|  | **Statistical Summary** | |  |  |  |  |  |  |
| **S.S** | 94.676 | 77.820 | 90.987 | 65.149 | 83.349 | 81.769 | 59.049 | 89.787 |
| **M.S** | 47.34 | 38.91 | 45.49 | 33.57 | 41.67 | 40.88 | 29.53 | 44.89 |
| **F** | 66.673*** | 28.54*** | 82.88*** | 18.59*** | 38.77*** | 28.75*** | 32.53*** | 52.88*** |
| **Df** | 2 | 2 | 2 | 2 | 2 | 2 | 2 | 2 |

Data in the columns presented as mean values ± standard error with various superscripts are significantly different according to DMRT（P > 0.05 ). S.S (Sum of square); M.S (Mean square); Df (Degree of freedom); F (Significance); CK (Check); *** (level of significance).


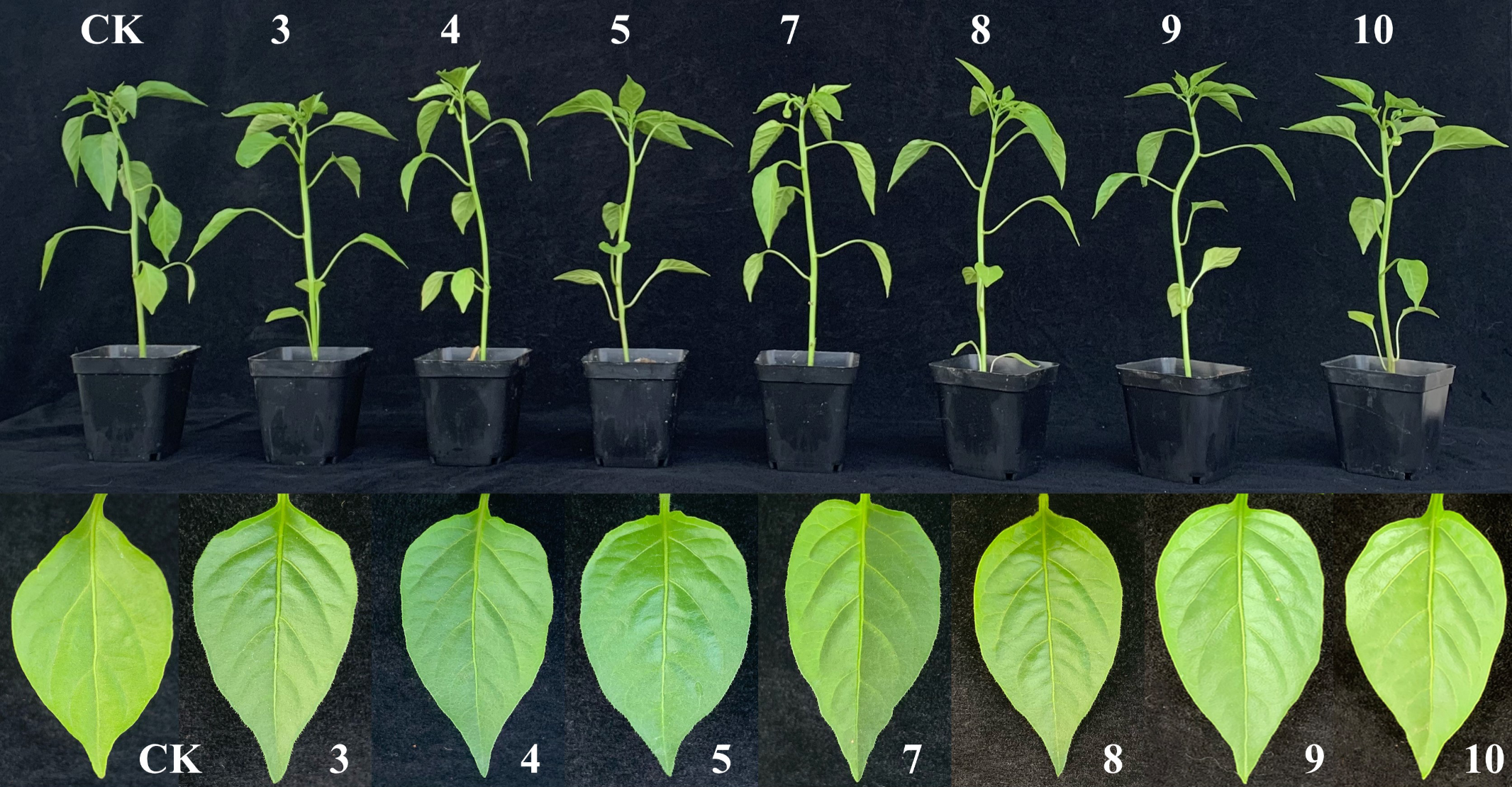


**Figure S43**. Effects of active compounds on pepper at 100 µg/mL

**Table S13.** Effects of active compounds on pepper height at 100 µg/mL

| **Day** | **Compound 3** | **Compound 4** | **Compound 5** | **Compound 7** | **Compound 8** | **Compound 9** | **Compound 10** | **CK** |
| --- | --- | --- | --- | --- | --- | --- | --- | --- |
| **0** | 16.53±0.98^c^ | 16.83±0.32^c^ | 16.40±0.53^c^ | 16.73±0.68^c^ | 17.03±0.56^c^ | 16.80±0.61^c^ | 16.86±0.84^c^ | 17.43±0.58^c^ |
| **7** | 21.50±0.75^b^ | 22.13±1.35^b^ | 20.57±0.87^b^ | 21.37±0.55^b^ | 20.90±0.93^b^ | 21.67±0.97^b^ | 23.26±0.55^b^ | 22.86±0.35^b^ |
| **14** | 27.50±0.61^a^ | 28.80±0.72^a^ | 28.20±0.78^a^ | 27.70±0.58^a^ | 28.23±0.59^a^ | 27.00±0.36^a^ | 28.60±0.36^a^ | 27.86±0.74^a^ |
|  | **Statistical Summary** | |  |  |  |  |  |  |
| **S.S** | 180.9 | 215.7 | 179.7 | 181.8 | 194.2 | 156.2 | 207.1 | 163.4 |
| **M.S** | 90.4 | 107.9 | 91.8 | 90.9 | 97.1 | 78.1 | 103.6 | 81.7 |
| **F** | 47.3*** | 43.9*** | 1.2*** | 83.5*** | 64.5*** | 54.2*** | 90.4*** | 80.9*** |
| **Df** | 2 | 2 | 2 | 2 | 2 | 2 | 2 | 2 |

Data in the columns presented as mean values ± standard error with various superscripts are significantly different according to DMRT（P > 0.05 ). S.S (Sum of square); M.S (Mean square); Df (Degree of freedom); F (Significance); CK (Check); *** (level of significance).


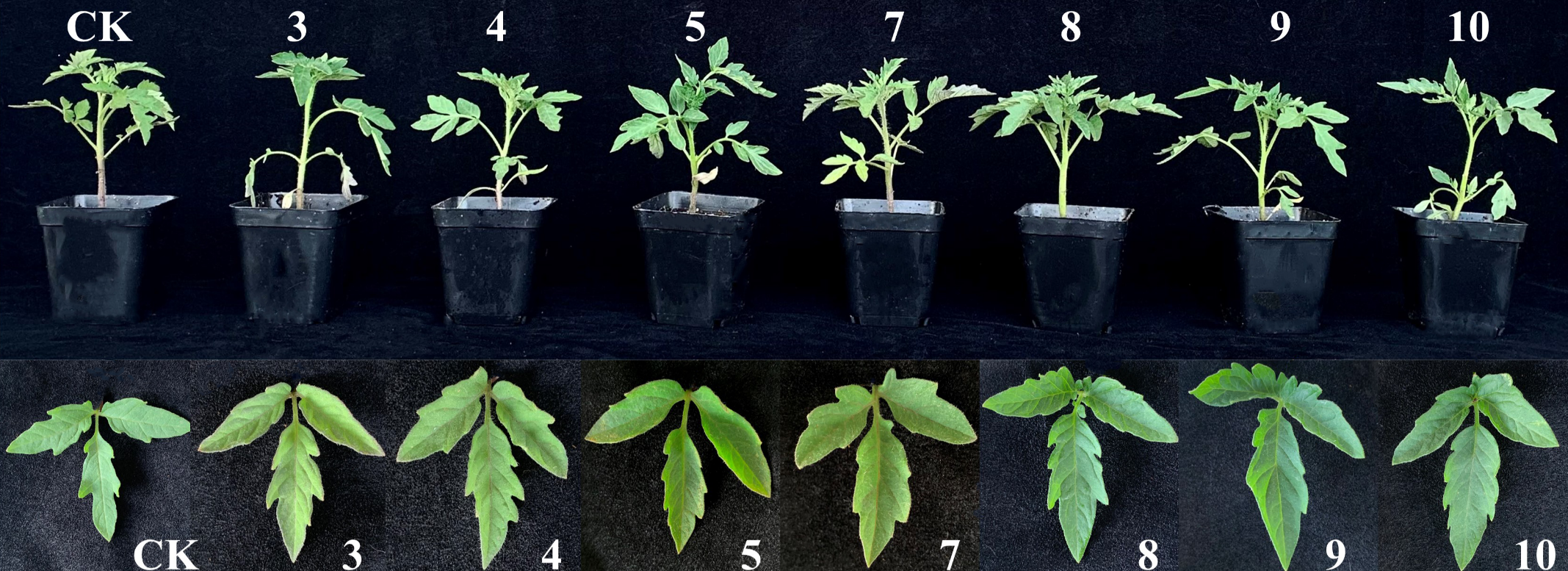


**Figure S44.** Effects of active compounds on tomato at 100 µg/mL

**Table S14.** Effects of active compounds on tomato height at 100 µg/mL

| **Day** | **Compound 3** | **Compound 4** | **Compound 5** | **Compound 7** | **Compound 8** | **Compound 9** | **Compound 10** | **CK** |
| --- | --- | --- | --- | --- | --- | --- | --- | --- |
| **0** | 10.47±04.^c^ | 10.36±0.6^c^ | 10.33±0.9^b^ | 9.3±0.6^a^ | 10.23±0.5^c^ | 10.13±0.2^c^ | 10.53±0.5^b^ | 10.5±0.6^c^ |
| **7** | 13.17±0.2^b^ | 12.80±0.5^b^ | 12.00±0.2^ab^ | 12.60±0.5^a^ | 12.83±0.9^b^ | 13.23±0.9^b^ | 12.70±0.9^b^ | 13.3±0.7^b^ |
| **14** | 15.83±0.6^a^ | 15.63±0.6^a^ | 15.20±1.4^a^ | 15.53±0.7^a^ | 16.13±0.8^a^ | 16.20±0.3^a^ | 15.80±0.9^a^ | 16.0±0.2^a^ |
|  | **Statistical Summary** | |  |  |  |  |  |  |
| **S.S** | 43.202 | 41.687 | 36.702 | 43.762 | 52.460 | 55.816 | 42.042 | 45.402 |
| **M.S** | 21.601 | 20.84 | 18.35 | 21.56 | 26.23 | 27.91 | 21.02 | 27.70 |
| **F** | 31.16** | 22.12** | 6.51*** | 1.02 NS | 16.16*** | 28.75*** | 11.25** | 23.79*** |
| **Df** | 2 | 2 | 2 | 2 | 2 | 2 | 2 | 2 |

Data in the columns presented as mean values ± standard error with various superscripts are significantly different according to DMRT（P > 0.05 ). S.S (Sum of square); M.S (Mean square); Df (Degree of freedom); F (Significance); CK (Check); **, *** (level of significance).


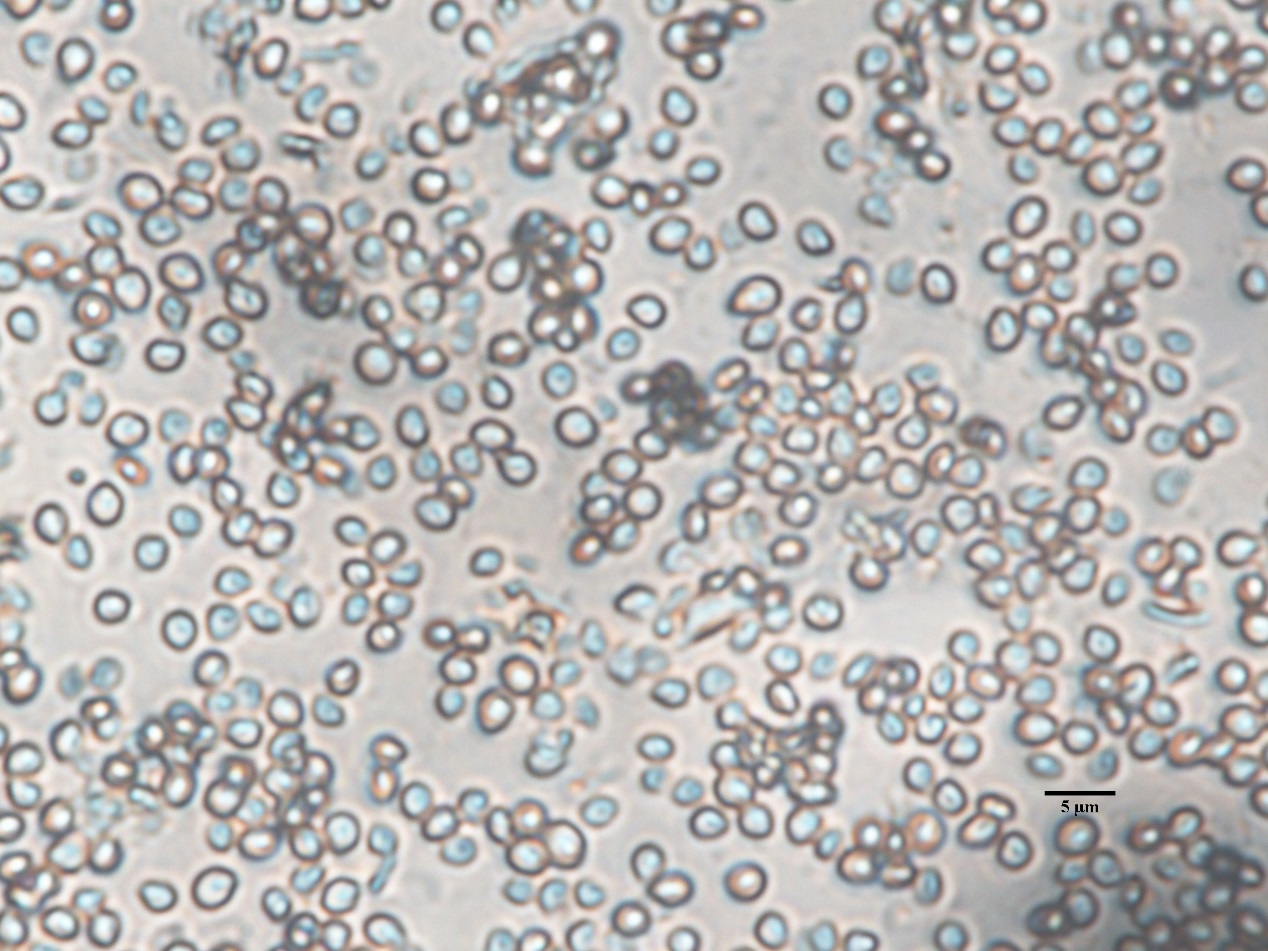


**Figure S45.** The conidia of *Beauveria* sp.

The conidia are spherical to nearly spherical, or oval-shaped, colorless and transparent, with thin walls. The size of the conidia is around 2. 41 (1.53- 3.26) μm × 2.62 (1.56-3.11) μm.
